# Global prevalence of mutation in the *mgrB* gene among clinical isolates of colistin-resistant *Klebsiella pneumoniae*: a systematic review and meta-analysis

**DOI:** 10.3389/fmicb.2024.1386478

**Published:** 2024-06-07

**Authors:** Amin Khoshbayan, Negar Narimisa, Zahra Elahi, Narjess Bostanghadiri, Shabnam Razavi, Aref Shariati

**Affiliations:** ^1^Microbial Biotechnology Research Center, Iran University of Medical Sciences, Tehran, Iran; ^2^Department of Microbiology, School of Medicine, Iran University of Medical Sciences, Tehran, Iran; ^3^Vice Chancellery of Education and Research, Torbat Heydariyeh University of Medical Sciences, Torbat Heydariyeh, Iran; ^4^Molecular and Medicine research center, Khomein University of Medical Sciences, Khomein, Iran; ^5^Infectious Diseases Research Center (IDRC), Arak University of Medical Sciences, Arak, Iran

**Keywords:** colistin, *mgrB*, *Klebsiella pneumoniae*, colistin-resistant, global prevalence

## Abstract

**Background:**

Colistin is used as a last resort for managing infections caused by multidrug-resistant bacteria. However, the high emergence of colistin-resistant strains has restricted the clinical use of this antibiotic in the clinical setting. In the present study, we evaluated the global prevalence of the mutation in the *mgrB* gene, one of the most important mechanisms of colistin resistance in *Klebsiella pneumoniae*.

**Methods:**

Several databases, including Scopus, Medline (via PubMed), and Web of Science, were searched (until August 2023) to identify those studies that address the *mgrB* mutation in clinical isolates of *K. pneumoniae*. Using Stata software, the pooled prevalence of *mgrB* mutation and subgroup analyses for the year of publication, country, continent, *mgrB* mutation types, and detection methods of *mgrB* mutation were analyzed.

**Results:**

Out of the 115 studies included in the analysis, the prevalence of *mgrB* mutations in colistin-resistant *K. pneumoniae* isolates was estimated at 65% of isolates, and *mgrB* variations with insertional inactivation had the highest prevalence among the five investigated mutations with 69%. The year subgroup analysis indicated an increase in mutated *mgrB* from 46% in 2014 to 61% in 2022. Europe had the highest prevalence of mutated *mgrB* at 73%, while Africa had the lowest at 54%.

**Conclusion:**

Mutations in the *mgrB* gene are reported as one of the most common mechanisms of colistin resistance in *K. pneumoniae,* and the results of the present study showed that 65% of the reported colistin-resistant *K. pneumoniae* had a mutation in this gene.

## 1 Introduction

The increasing prevalence of infections due to multidrug-resistant (MDR) bacteria is a major public health concern, and the emergence of antimicrobial resistance has created a difficult challenge for treating a wide variety of infectious diseases (Dadashi et al., 2022). Today, colistin is considered one of the last remaining options for physicians in the fight against MDR and pan-drug-resistant (PDR) bacteria (Moubareck et al., 2018; Menekşe et al., 2019; Moghadam et al., 2022). Colistin, or polymixin E, is a cationic antibiotic and belongs to the polymixin antibiotic class that has that have activity against most Gram-negative bacteria. In the past, colistin had limited use in medicine because of its toxicity, especially nephrotoxicity, but in recent years, due to the increasing rate of MDR bacteria, especially carbapenemase-producing strains, the application of colistin has become more common (Caniaux et al., 2017; Poirel et al., 2017).

However, the high prevalence of colistin-resistant (ColR) strains has restricted the clinical use of colistin. Moreover, a worrying 25–71% mortality rate is reported for colistin-resistant infections (Moubareck et al., 2018; Menekşe et al., 2019; Moghadam et al., 2022).

Enterobacteriaceae cause a wide range of infections in humans. They are capable of acquiring resistance to many antibiotics through horizontal gene transfer (Hasani et al., 2017; Dadashi et al., 2022). Among the bacteria in this family, *K. pneumoniae* is the most common species that has developed resistance to colistin. Colistin resistance in *K. pneumoniae* has been reported worldwide in Asia, Europe, North America, South America, and Africa (Ah et al., 2014; Giamarellou, 2016).

Furthermore, resistance to colistin is mainly mediated through chromosomes or horizontal gene transfer. For the first time, the plasmid-borne *mcr-1* gene was reported from China, and to date, 10 different types of *mcr* genes have been reported (Liu et al., 2016; Caniaux et al., 2017; Aris et al., 2020; Hussein et al., 2021). Additionally, chromosomal gene mutations such as *pmrA/pmrB*, *crrA/crrB*, and *phoP/phoQ*, as well as variations in *mgrB*, are believed to be significant factors in the development of colistin resistance in *K. pneumoniae* (Cannatelli et al., 2014; Poirel et al., 2017).

The PmrAB and PhoPQ two-component systems are associated with bacterial survival and are usually activated when macrophages attack bacteria. The Pmr system consists of genes and operons involved in adding phosphoethanolamine and 4-amino-4-deoxy-L-arabinose to lipopolysaccharide (LPS; Gunn, 2008; Poirel et al., 2017).

To this end, the inactivation of *mgrB* causes a negative feedback regulator of the PhoQ-PhoP signaling system, which leads to the acquisition of colistin resistance in *K. pneumoniae*. This phenomenon ultimately activated the Pmr system, causing modification and reduced affinity of the LPS, which is the colistin target (Cannatelli et al., 2013; Khoshbayan et al., 2021). Collectively, *mgrB* variation is reported as one of the most common resistance mechanisms among ColR *K. pneumoniae* isolates (Aghapour et al., 2019). However, there is no exact report on its prevalence among clinical isolates of *K. pneumoniae*. Therefore, this study aims to investigate the global prevalence of the mutation in the *mgrB* among clinical isolates of ColR *K. pneumoniae*.

## 2 Methods

### 2.1 Search strategy

A comprehensive and systematic search was conducted for relevant articles by two authors (AKH and NB) until August 2023 in the electronic databases, including Medline (via PubMed), Scopus, and Web of Science. The following search keywords were obtained from the National Library of Medicine’s medical subject heading (MeSH) terms, titles, or abstracts with the help of Boolean operators (and/or) including “*Klebsiella pneumoniae*” AND “*mgrB*” with their Mesh terms. The present study was conducted according to the Preferred Reporting Items of the Systematic Review and Meta-Analysis (PRISMA) guidelines.

### 2.2 Selection criteria and data extraction

Two authors (AKH and NB) worked independently to review the titles, abstracts, and full texts of all retrieved studies, and they excluded irrelevant articles (review articles, case reports, short communication, letters to the editor, brief reports, conference abstracts, and studies with ambiguous results). The search was limited to articles published in English that reported the prevalence of the *mgrB* in clinical isolates of ColR *K. pneumoniae*. Disagreements among authors were resolved through discussion and consensus. The information extracted from each of the included articles is as follows: first author name, publication year, country, continent, the total number of *K. pneumoniae* isolates, number of ColR isolates, number of ColR isolates carrying the mutated *mgrB*, the *mgrB* mutation types, and method used for detection of *mgrB* mutation.

### 2.3 Quality assessment

An adapted version of the Joanna Briggs Institute (JBI) checklist was used to independently assess study quality by two review authors (ZE and NN; Moola et al., 2017).

### 2.4 Statistical analysis

A meta-analysis was performed using Stata software v. 17, and a random-effects model estimated the pooled prevalence of the mutated *mgrB* in ColR *K. pneumoniae* isolates and the prevalence of five types of *mgrB* mutation (insertional inactivation, substitution, nonsense mutation, complete and partial deletion) with 95% confidence intervals (95% CI). A Freeman-Tukey double arcsine transformation was performed using the metaprop command of Stata software to estimate the weighted pooled fractions. The I^2^ value was used to examine statistical heterogeneity between studies. In this regard, I^2^ ≤ 25% was considered low homogeneity, 25% < I^2^ ≤ 75% shows moderate heterogeneity, and I^2^ > 75% indicates high heterogeneity. Potential publication bias was checked using funnel plots and Begg tests. Subgroup analyses were performed for the year of publication, country, continent, and methods used to detect *mgrB* variations.

## 3 Results

### 3.1 Search results

A total of 769 studies were identified in the three electronic databases up to August 2023, and 592 articles were included after duplicate removal. 258 studies after an initial screening of the title and abstract, were eligible for further analysis, of which 115 were included in the final analysis (Supplementary 2, Figure 1).

### 3.2 Meta-analysis results

In the 115 studies, 2,652 ColR *K. pneumoniae* and 1,448 ColR isolates with a change in *mgrB* were found (Table 1). The pooled prevalence of *mgrB* variations in ColR *K. pneumoniae* isolates was detected in 65% of isolates (95% CI: 56–72%; I ^2^ = 91.67%; *p* < 0.001; Supplementary File 3). The results of Begg’s test (*p* = 0.4202) showed no publication bias in our study. Noteworthy, the result of publication bias was shown in the funnel plot (Supplementary 2, Figure 2). The year subgroup analysis indicated an increase in mutated *mgrB* from 46% (95% CI: 27–65%) in 2014 to 61% (95% CI: 43–78%) in 2022. However, in 2023, the results showed a decrease in the rate of mutation to 39% (95% CI: 5–80%), which could be due to the small number of studies compared to 2022 (*p* = 0.259; Supplementary 2, Figure 3). A subgroup meta-analysis of continents also showed that Europe had the highest rate of mutated *mgrB* (73%; 95% CI: 63–82%), while Africa had the lowest rate (54%; 95% CI: 9–96%; *p* = 0.445; Supplementary 2, Figures 4, 5). Among the countries analyzed, Tunisia (95% CI: 97–100%) and Israel (95% CI: 80–100%) with 100% had the highest prevalence of mutated *mgrB,* while Spain with 8% (95% CI: 0–33%) showed the lowest (*p* < 0.001; Supplementary 2, Figure 6). Subgroup meta-analysis based on the detection method of mutated *mgrB* revealed 59% (95% CI: 49–69%) for the polymerase chain reaction (PCR) method and 71% (95% CI: 57–84%) for the whole genome sequencing (WGS) method (*p* = 0. 219; Supplementary 2, Figure 7). The pooled prevalence of *mgrB* variations with insertional inactivation in the total number of *mgrB* variations of ColR *K. pneumoniae* isolates was 69% (95% CI: 56–72%; I^2^ = 79.37%; *p* < 0.001; Supplementary 2, Figure 8). The results of the subgroup meta-analysis showed the only significant difference in the subgroup of countries. Spain had the highest mutation rate with 100% (95% CI: 57–100%) and Serbia had the lowest mutation rate with 0.0% (95% CI: 0–4%), (*p* < 0.001; Supplementary 4, Figure 3). The pooled prevalence of *mgrB* variations with substitution in the total number of *mgrB* variations of ColR *K. pneumoniae* isolates was 36% (95% CI: 25–48%; I^2^ = 87.31%; *p* < 0.001; Supplementary 2, Figure 9). The results of the subgroup meta-analysis showed an increase in the substitution mutation from 18% (95% CI: 8–30%) in 2014 to 50% (95% CI: 19–81%) in 2022 (*p* < 0.001; Supplementary 4, Figure 5). The highest prevalence of substitution mutation was observed in Brazil at 73% (95% CI: 4–100%), while Taiwan and Greece had the lowest rates with 11% each (95% CI: 2–24% and 6–18%, respectively; *p* = 0.003; Supplementary 4, Figure 7). Moreover, the subgroup meta-analysis based on the diagnostic method revealed that WGS detected the mutations in 60% of cases (95% CI: 39–80%), while PCR detected mutations in 16% of cases (95% CI: 10–24%; *p* < 0.001; Supplementary 4, Figure 8). The pooled prevalence of *mgrB* variations with nonsense mutations in the total number of *mgrB* variations of ColR *K. pneumoniae* isolates was 30% (95% CI: 19–42%; I^2^ = 88.63%; *p* < 0.001; Supplementary 2, Figure 10). The results of the subgroup meta-analysis showed an increase in nonsense mutations from 18% (95% CI: 9–29%) in 2014 to 100% (95% CI: 100–100%) in 2023 (*p* < 0.001; Supplementary 4, Figure 9). In addition, Asia had the highest rate of nonsense mutation with 36% (95% CI: 19–55%), while South America had the lowest rate with only 7% (95% CI: 1–17%; *p* < 0.001; Supplementary 4, Figure 10). Of the countries studied, Iran had the highest prevalence of nonsense mutation, which was 69% (95% CI: 49–87%). On the other hand, Brazil and Serbia had the lowest rate of this mutation, which was 8% (95% CI: 1–18%) and 8% (95% CI: 0–22%), respectively (*p* < 0.001; Supplementary 4, Figure 11). The pooled prevalence of *mgrB* variations with complete deletion in the total number of *mgrB* variations of ColR *K. pneumoniae* isolates was 19% (95% CI: 11–28%; I^2^ = 56.99%; *p* < 0.001; Supplementary 2, Figure 11). The results of the subgroup meta-analysis showed an increase in complete deletion in *mgrB* from 9% (95% CI: 1–21%) in 2014 to 30% (95% CI: 13–49%) in 2022 (*p* = 0.002; Supplementary 4, Figure 13). Furthermore, the pooled prevalence of *mgrB* variations with partial deletion in the total number of *mgrB* variations of ColR *K. pneumoniae* isolates was 14% (95% CI: 6–22%; I^2^ = 69.78%; *p* < 0.001; Supplementary 2, Figure 12). Among the countries investigated, Brazil had the highest prevalence of partial deletion in *mgrB* with 52% (95% CI: 9–94%), while Taiwan had the lowest rate of this mutation with 6% (95% CI: 1–14%; *p* = 0.003; Supplementary 4, Figure 18).

**Table 1:** Characteristics of included studies that reported resistance to colistin by *mgrB* mutation in the present meta-analysis.

Author and references	Year	Country	Continent	No. of *K. pneumoniae* isolates	Number of colistin-resistant isolates	Number of *mgrB* mutant isolates	Percentage of *mgrB* mutants in colistin-resistant isolates	Method	Mutation type
Abozahra et al. (2023)	2023	Egypt	Africa	82	32	4	13%	PCR	4 NM
Al-Farsi et al. (2019)	2019	Sweden	Europe	245	8	8	100%	PCR	8 II
Arena et al. (2022)	2022	Italy	Europe	19	7	2	29%	WGS	2 not report
Avgoulea et al. (2018)	2018	Greece_Italy	Europe	19	19	19 (10)	100%	WGS	10 II
Azam et al. (2021)	2021	India	Asia	335	11	4	36%	PCR	3 II, 1 S
Baron et al. (2021)	2020	France	Europe	5,304	14	2	14%	WGS	1 II, 1 NM
Barragán-Prada et al. (2019)	2019	Spain	Europe	30	21	3	14%	PCR	3 II
Bathoorn et al. (2016)	2016	Greece	Europe	34	19	17	89%	WGS	3 S, 14 II
Becker et al. (2018)	2018	Germany	Europe	53	1	1	100%	WGS	1 NM
Ben-Chetrit et al. (2021)	2021	Israel	Asia	7	6	6	100%	WGS	2 II, 1 PD, 2 CD, 1 NM
Ben Sallem et al. (2022)	2022	Tunisia	Africa	25	1	1	100%	PCR	1 S
Zahedi Bialvaei et al. (2023)	2023	Iran	Asia	162	161	2	1%	PCR	2 NM
Bir et al. (2022)	2022	India	Asia	48	7	2	29%	WGS	2 S
Bolourchi et al. (2021)	2021	Iran	Asia	138	14	6	43%	WGS	1 II, 2 NM, 2 S, 1 PD
Bonura et al. (2015)	2015	Italy	Europe	94	39	31	79%	PCR	13 NM, 16 II, 2 S
Boszczowski et al. (2019)	2019	Brazil	South America	28	26	5	19%	WGS	4 S, 1 not report
Cabanel et al. (2021)	2021	France_Spain	Europe	18	1	1	100%	WGS	1 CD
Can et al. (2018)	2018	Turkey	Europe	115	115	83	72%	PCR	77 II, 6 point mutation and deletions
Cannatelli et al. (2014)	2014	Italy-Greece	Europe	66	66	39	59%	PCR	22 II, 4 CD, 6 NM, 7 S
Cejas et al. (2019)	2019	Argentina	South America	76	11	7	64%	PCR	4 CD, 1 NM, 2 S
Chen et al. (2021)	2021	China	Asia	3	2	2	100%	WGS	2 II
Chen et al. (2022)	2022	China	Asia	493	11	8	73%	WGS	7 II, 1 PD
Cheng et al. (2015)	2015	Taiwan	Asia	26	26	10	38%	PCR	8 II, 2 deletion
Cheong et al. (2020)	2020	Korea	Asia	252	11	6	55%	PCR	5 S, 1 II
Cienfuegos-Gallet et al. (2017)	2017	Colombia	South America	156	32	24	75%	PCR	22 II, 1 NM, 1 frameshift
Conceição-Neto et al. (2022)	2022	Brazil	South America	502	148	39	26%	PCR	28 II, 1 S and NM, 8 S, 2 NM
Di Pilato et al. (2021)	2020	Italy	Europe	156	63	56	89%	WGS	5 S and NM, 2 NM, 19 II, 25 S, 5 PD
Di Tella et al. (2019)	2019	Italy	Europe	26	19	19	100%	PCR	9 S, 6 II, 2 PD, 1 NM, 1 not reported
Dong et al. (2018)	2018	China	Asia	5	2	2	100%	WGS	2 II
D’Onofrio et al. (2020)	2020	Croatia	Europe	6	6	3	50%	WGS	1 II, 2 S
Elias et al. (2022)	2022	Portugal	Europe	140	16	8	50%	PCR	2 NM, 1 S, 3 II, 2 CD
Esposito et al. (2018)	2018	Italy	Europe	25	25	22	88%	PCR	5 II, 10 PD, 3 S, 4 NM
Főldes et al. (2022)	2022	Romania	Europe	10	10	7	70%	WGS	3 II, 4 S
Garcia-Fulgueiras et al. (2021)	2020	Uruguay	South America	3	2	2	100%	WGS	2 II
Garza-Ramos et al. (2023)	2022	Mexico	South America	101	18	1	6%	PCR	1 II
Gentile et al. (2020)	2020	Italy	Europe	27	27	13	48%	WGS	8 PD,1 CD, 1 II, 3 S
Haeili et al. (2017)	2017	Iran	Asia	20	20	15	75%	PCR	6 II, 9 NM
Halaby et al. (2016)	2016	Netherlands	Europe	8	2	1	50%	WGS	1 II
Hamel et al. (2020)	2020	Greece	Europe	973	213	148	69%	PCR	94 II, 24 S, 4 NM, 21 CD, 5 PD
Hu et al. (2023)	2023	China	Asia	708	14	9	64%	WGS	3 CD, 6 II
Huang et al. (2021)	2021	Taiwan	Asia	229	24	17	71%	PCR	10 II, 1 NM, 1 PD, 1 S, 4 Not detected
Huang et al. (2022)	2022	Taiwan	Asia	35	35	18	51%	PCR	3 S, 9 II, 1 PD, 2 frameshift, 3 not detected
Jaidane et al. (2018)	2017	Tunisia	Africa	2,826	13	13	100%	WGS	2 S and II, 5 S, 1 CD, 2 PD, 3 S and PD
Jayol et al. (2016)	2016	France	Europe	561	35	17	49%	PCR	10 II, 2 NM, 2 CD, 2 PD, 1 S
Jayol et al. (2018)	2018	Switzerland_France	Europe	46	35	17	49%	PCR	2 S, 3 NM, 1 PD, 11 II
Jin et al. (2021)	2021	China	Asia	11	4	2	50%	WGS	2 NM
Karampatakis et al. (2022)	2022	Greece	Europe	4	4	4	100%	PCR	4 II
Kaza et al. (2024)	2023	India	Asia	775	18	7	39%	WGS	5 II, 1 S, 1 PD
Khoshbayan et al. (2022)	2022	Iran	Asia	195	21	19	90%	PCR	19 II
Kim et al. (2020)	2019	Korea	Asia	25	4	4	100%	WGS	4 II
Kis et al. (2016)	2016	Hungry	Europe	312	3	3	100%	PCR	3 II
Kong et al. (2021)	2021	China	Asia	2	1	1	100%	WGS	1 NM
Kumar et al. (2018)	2018	India	Asia	932	17	4	24%	PCR	3 II, 1 NM
Lalaoui et al. (2019)	2018	Israel	Asia	15	3	3	100%	PCR	1 II, 2 S
Lee et al. (2021)	2021	Korea	Asia	338	2	2	100%	PCR	2 II
Leung et al. (2017)	2017	USA	North America	22	11	8	73%	PCR	1 S, 3 II, 1 NM, 2 deletion, 1 frameshift
Liu et al. (2022)	2022	China	Asia	1884	14	7	50%	WGS	1 S, 5 II, 1 NM
Lomonaco et al. (2018)	2018	Pakistan-USA	Asia-North America	10	7	4	57%	WGS	3 II, 1 CD
Longo et al. (2019)	2019	Brazil	South America	23	23	7	30%	WGS	4 II, 3 PD
López-Camacho et al. (2014)	2013	Spain	Europe	26	1	1	100%	WGS	1 II
Malli et al. (2018)	2018	Greece	Europe	131	98	75	77%	PCR	36 II, 22 NM, 6 S, 11 deletion
Mansour et al. (2017)	2017	Tunisia	Africa	220	7	7	100%	PCR	7 II
Markovska et al. (2022)	2022	Bulgaria	Europe	100	29	9	31%	PCR	5 II, 2 NM, 2 not detected
Mathur et al. (2018)	2018	India	Asia	8	8	2	25%	WGS	2 S
Mavroidi et al. (2016)	2016	Greece	Europe	135	19	15 (2)	79%	PCR	2 II
Mavroidi et al. (2020)	2019	Greece	Europe	53	28	15 (4)	54%	PCR	4 II
Mills et al. (2021)	2021	USA	North America	27	7	5	71%	WGS	2 NM, 2 II, 1 S
Mirshekar et al. (2020)	2020	Iran	Asia	94	20	4	20%	PCR	3 NM, 1 II
Moghimi et al. (2021)	2021	Iran	Asia	5	2	2	100%	PCR	2 NM
Naha et al. (2022)	2022	India	Asia	240	9	3	33%	WGS	2 S, 1 NM
Nawfal Dagher et al. (2019)	2019	Lebanon	Asia	5	2	1	50%	PCR	1 S
Ngbede et al. (2021)	2021	Nigeria-USA	Africa-North America	16	16	16	100%	WGS	16 S
Nguyen et al. (2021)	2021	Vietnam	Asia	8	3	3	100%	WGS	3 II
Niazadeh et al. (2022)	2022	Iran	Asia	65	6	5	83%	PCR	4 S, 1 deletion
Nirwan et al. (2021)	2021	India	Asia	16	13	3	23%	PCR	1 S, 2 II
Nordmann et al. (2016)	2016	Switzerland	Europe	121	94	64	68%	PCR	7 S, 11 NM, 33 II, 4 CD, 8 PD, 1 PD and S
Novović et al. (2017)	2017	Serbia	Europe	27	27	2	7%	PCR	1 II, 1 NM
Okdah et al. (2022)	2022	Saudi Arabia	Asia	10	10	4	40%	WGS	2 S, 2 inactivation
Olaitan et al. (2014)	2014	-	-	32	32	13	41%	WGS	3 NM, 3 S, 5 II, 2 not detected
Otter et al. (2017)	2017	UK	Europe	38	25	23	92%	WGS	23 NM
Palani et al. (2020)	2020	India	Asia	-	25	11	44%	PCR	8 CD, 1 NM, 2 II
Palmieri et al. (2020)	2020	Serbia	Europe	2,298	45	45	100%	WGS	38 S, 6 NM, 1 II
Pitt et al. (2018)	2018	Australia	Oceania	24	19	17	89%	PCR-WGS	14 II, 2 NM, 1 S
Poirel et al. (2015)	2014	-	-	47	47	12	26%	PCR	9 II, 3 NM
Popa et al. (2021)	2021	Romania	Europe	23	1	1	100%	WGS	1 NM
Pragasam et al. (2017)	2021	India	Asia	8	8	4	50%	PCR	2 NM, 2 PD
Pu et al. (2023)	2023	China	Asia	12	3	2	67%	WGS	2 II
Rimoldi et al. (2017)	2017	Italy	Europe	68	7	2	29%	WGS	2 II
Roch et al. (2022)	2022	Brazil	South America	43	35	35	100%	WGS	35 S
Rocha et al. (2020)	2020	Brazil	South America	2	2	1	50%	WGS	1 II
Rocha et al. (2022)	2022	Brazil	South America	56	56	49 (13)	88%	PCR	9 II, 3 NM, 1 PD
Rubic et al. (2023)	2023	Croatia	Europe	34	34	34	100%	PCR	34 NM
Shamina et al. (2020)	2020	Russia	Europe	159	71	23	32%	PCR	19 II, 4 CD
Shankar et al. (2019)	2019	India	Asia	65	65	13	20%	PCR	3 NM, 6 II, 3 S, 1 No amplification
Sharahi et al. (2021)	2021	Iran	Asia	52	16	6	38%	PCR	5 NM, 1 II
Singh et al. (2021)	2021	India	Asia	22	22	3	14%	PCR	3 II
Sisti et al. (2022)	2022	Italy	Europe	12	4	3	75%	PCR	1 NM, 1 CD, 1 PD
Snyman et al. (2021)	2021	South Africa	Africa	7	7	2	29%	WGS	1 CD, 1 II
Solgi et al. (2020)	2020	Iran	Asia	74	1	1	100%	PCR	1 II
Sonnevend et al. (2017)	2017	UAE	Asia	9	9	9	100%	PCR	9 II
Tietgen et al. (2022)	2022	Germany	Europe	12	12	10	83%	PCR	5 II, 5 CD
Torres et al. (2021)	2021	Switzerland	Europe	20	11	10	91%	WGS	2 II, 4 NM, 4 S
Zaman et al. (2018)	2018	Saudi Arabia	Asia	23	23	18	78%	PCR	17 II, 1 NM
Vendrik et al. (2022)	2022	Netherlands	Europe	36	18	7	39%	NGS	1 PD, 3 II, 2 S, 1 CD
Wang et al. (2023)	2023	China	Asia	189	4	2	50%	NGS	2 II
Wright et al. (2015)	2014	USA	North America	11	9	6	67%	RNA-Seq	1 S, 4 II, 1 CD
Xiao et al. (2023)	2023	China	Asia	458	28	1	4%	WGS	1 S
Xie et al. (2022)	2022	China	Asia	2	1	1	100%	ND	1 II
Yang et al. (2020)	2020	Taiwan	Asia	49	49	32	65%	PCR	6 NM, 17 II, 6 CD, 2 PD, 1 isolate with different pattern
Yap et al. (2020)	2019	Malaysia	Asia	2	2	2	100%	WGS	2 II
Yoshino et al. (2021)	2021	Japan	Asia	5	1	1	100%	WGS	1 CD
Yousfi et al. (2019)	2018	Algeria	Africa	3	3	1	33%	PCR	1 II
Zafer et al. (2019)	2019	Egypt	Africa	234	22	1	5%	PCR	1 S
Zhang et al. (2018)	2018	China	Asia	17	8	8	100%	WGS	8 II
Zhu et al. (2019)	2019	Greece	Europe	16	8	8	100%	PCR	8 II

PD, Partial Deletion; CD, Complete Deletion; II, Insertional Inactivation; NM, Nonsense Mutations; S, Substitution; ND, Not Determined. In four studies number of *mgrB* mutated isolates were different from number of *mgrB* detected isolates that written in parentheses.

## 4 Discussion

In recent years, the effectiveness of antibiotics against MDR pathogens has decreased, leaving colistin as the last available option (Lim et al., 2010). Numerous mechanisms in Gram-negative bacteria result in changes to the outer membrane, which are the main causes of colistin resistance (Li et al., 2006). As mentioned, *mgrB* inactivation leads to dysregulation of the PhoQ-PhoP signaling system, eventually leading to LPS modification (Cannatelli et al., 2013).

A recent study declared that MgrB alteration could create a fitness cost in *K. pneumoniae* related to the bacteria’s environmental survival. This phenomenon could pose a silent threat to hospital transmission, as the physical changes resulting from the *mgrB* mutation seem to cause resistance to disinfectants.

Furthermore, during a two-year period, Xie et al. isolated one colistin-susceptible isolate and one *mgrB-mutated* ColR isolate from a patient. The ColR isolate exhibits an increased growth rate, but the colistin-susceptible isolate showed significantly decreased growth during a three-hour period, indicating that colistin resistance might result in resistance to human serum (Xie et al., 2022; Yap et al., 2022). Furthermore, the results of a recently published study showed that mutation of *mgrB* led to resistance to the *Galleria mellonella* antimicrobial peptides, and in both *in vivo* and *in vitro* experiments, it stimulated little activation of inflammatory responses. This phenomenon could be related to the increased virulence associated with this mutation, as many studies have shown the importance of an inflammatory response for *K. pneumoniae* clearance (Kidd et al., 2017). Interestingly, another study demonstrated that MgrB-dependent ColR *K. pneumoniae* isolates exhibit increased survival outside the host, leading to enhanced host-to-host transmission (Bray et al., 2022). Therefore, physicians and researchers must appreciate the importance of *mgrB* mutant isolates for cautious consideration of colistin utilization in *K. pneumoniae* infections. The significant rise in ColR isolates observed in recent years is related to the rapidly increasing use of colistin in hospital settings, which eventually accelerates the selection pressure for resistance (Wang et al., 2017; Liu and Liu, 2018). Nevertheless, the precise prevalence of *mgrB* variations was not reported in the recently published studies, therefore, the current study investigates the prevalence of mutated *mgrB* among the clinical isolates of ColR *K. pneumoniae* worldwide.

According to our analysis, 65% of all the ColR *K. pneumoniae* isolates carried mutated *mgrB*. Furthermore, the prevalence of the *mgrB* mutation has steadily increased from 46% in 2014 to 61% in 2022, which is a 15% increase. Similarly, a recent study demonstrates an increase in ColR from 4.8% in 2013–2018 to 8.2% in 2019–2021 in Iran (Narimisa et al., 2022). Moreover, the annual report of the European Antimicrobial Resistance Surveillance Network (EARS-Net) declared that ColR *K. pneumoniae* has reached a high level of more than 20% in Italy and Greece (Prevention ECfD, Control, 2017; Liu and Liu, 2018). The increasing global use of colistin could lead to an enhanced increase in resistance to the antibiotic, as shown by our analysis of a 15% increase. This phenomenon highlights the urgent need to evaluate the strategies of antimicrobial resistance management internationally (Yusof et al., 2022).

Our results showed that Europe showed the highest rate of mutated *mgrB* among the continents with 73%, and Africa had the lowest prevalence, with 54%. In 2012, Jaidane et al. demonstrated the emergence of colistin resistance in Tunisia and showed the critical role of MgrB in ColR *K. pneumoniae* isolates (Jaidane et al., 2018). Furthermore, of the 47 ColR *K. pneumoniae isolates* in Thailand, mutated *mgrB* was the leading cause of ColR, which was observed among 43 (91.5%) isolates (Shein et al., 2022). Moreover, a recently published study declared that the most common resistance mechanism among ColR *K. pneumoniae* isolates in the Middle East is mutations and insertion sequence transpositions in the *mgrB* (Aris et al., 2020). Moreover, a recent study investigating the prevalence of mutated ColR *K. pneumoniae* reported that four countries in the Middle East had a high prevalence (>50%) of mutated ColR *K. pneumoniae* (Saudi Arabia, Qatar, Tunisia, and Iran; Yusof et al., 2022). We observed various mutations in the *mgrB* locus and categorized them into five groups: insertional inactivation, substitution, nonsense mutation, complete deletion, and partial deletion To view the details, you can refer to the Supplementary Excel file. The prevalence of substitution and complete deletion increased from 2014 to 2022 from 18 to 50% and 9 to 30%, respectively. Additionally, the prevalence of nonsense mutations has increased from 18% in 2014 to 100% in 2023. Insertional inactivation had the highest pooled prevalence among the *mgrB* variations, at 69%. These small mobile genetic elements are found in the genomes of most bacteria and pose a severe danger to gene structure and expression (Consuegra et al., 2021).

The insertion of IS elements leads to the inactivation or truncation of *mgrB*, resulting in the malfunction of MgrB (Yang et al., 2020). On many occasions, IS elements are carried by Inc. plasmid groups, and some studies indicate that these plasmids may also carry other resistance genes, like carbapenemase (Fordham et al., 2022). The presence of multidrug-resistant IS-carrying plasmids is a significant concern. The emergence of antimicrobial resistance can lead to colistin therapy, which can mobilize IS elements and potentially create extensively drug-resistant (XDR) or PDR isolates (Fordham et al., 2022). Therefore, monitoring the mutations caused by IS elements in *K. pneumoniae* is crucial to prevent the worldwide spread of colistin resistance (Yang et al., 2020; Yusof et al., 2022).

Generally, in the analysis of detection methods, it was found that both PCR and WGS methods were equally effective in detecting mutations, with no clear superiority of one over the other. However, WGS was more effective in detecting substitution mutations in 60% of cases, while PCR was effective only in 16%. Therefore, WGS can be considered to be the ideal method for detecting this specific mutation. In combination with Sanger sequencing, PCR has been traditionally used as the gold standard for mutation detection for many years due to its high specificity and low rate of false positives. Although this method has some limitations, such as low sensitivity, it is also time-consuming because of the need for manual analysis of sequencing chromatograms (Gao et al., 2016). Despite these limitations, due to its accessibility and low cost, PCR is still a reasonable and affordable method, especially in developing countries.

## 5 Limitations

Our study has certain limitations. Because only one study was conducted on the Oceania continent, we could not compare the prevalence of the *mgrB* mutation in ColR *K. pneumoniae* with other continents. We did not investigate the sequence type (ST) of resistant isolates because some studies did not report or determine the ST type. In addition, the heterogeneity among studies was relatively high; therefore, subgroup analysis was used to find and reduce the source of heterogeneity.

## 6 Conclusion

Given the high importance and rise in the global prevalence of ColR *K. pneumoniae* isolates, it is vital to know the underlying mechanisms related to colistin resistance. The results of the present study showed that 65% of the ColR *K. pneumoniae* had variation in this gene. Collectively, these findings emphasize the importance of regular monitoring of ColR isolates in clinical settings to stop the spread of ColR isolates. Additionally, adopting innovative screening techniques, practicing antibiotic stewardship, lowering the usage of antibiotics in agriculture, and emphasizing the urgent need to design an organized plan to measure the colistin resistance level are effective strategies to combat antibiotic resistance. In this concept, the exact detection of mechanisms that lead to the mutation in *mgrB* could significantly decrease the extension of ColR *K. pneumoniae*. However, more confirmatory studies are needed to advance our knowledge in this field.

## Data availability statement

The datasets presented in this study can be found in online repositories. The names of the repository/repositories and accession number(s) can be found in the article/Supplementary material.

## Author contributions

AK: Investigation, Writing – original draft, Writing – review & editing. NN: Writing – original draft, Writing – review & editing. ZE: Writing – review & editing. NB: Writing – review & editing. SR: Writing – review & editing. AS: Writing – review & editing.
