# Supplementary material for: Global prevalence of mutation in the mgrB gene among clinical isolates of colistin-resistant Klebsiella pneumoniae: a systematic review and meta-analysis
Source: Front Microbiol. 2024 Jun 7;15:1386478. doi: 10.3389/fmicb.2024.1386478 (PMC11190090; doi:10.3389/fmicb.2024.1386478)
Supplement: Supplementary file 2 [file Data_Sheet_1.PDF]

First Author Name of Study	Q1	Q2	Q3	Q4	Q5	Q6	Q7	Q8	Q9	Total
Abozahra	Y	Y	Y	Y	N	Y	Y	N	Y	7
Al_Farsi	Y	Y	Y	Y	Y	Y	Y	Y	Y	9
Arena	Y	Y	Y	Y	Y	Y	Y	Y	Y	9
Avgoulea	Y	Y	Y	Y	Y	Y	Y	N	Y	8
Azam	Y	Y	Y	Y	Y	Y	Y	U	Y	8
Baron	Y	Y	Y	Y	Y	Y	Y	Y	N	8
Barragán_Prada	Y	Y	Y	Y	Y	Y	Y	N	Y	8
Bathoorn	Y	Y	Y	Y	N	Y	Y	N	Y	8
Becker	Y	Y	Y	Y	N	Y	Y	N	Y	7
Ben_Chetrit	Y	Y	Y	N	N	Y	Y	N	Y	6
Ben_Sallem	Y	Y	Y	Y	Y	Y	Y	N	Y	8
Bialvaei	Y	Y	Y	Y	Y	Y	Y	Y	Y	9
Bir	Y	Y	Y	N	N	Y	Y	Y	Y	7
Bolourchi	Y	Y	Y	Y	Y	Y	Y	N	Y	8
Bonura	Y	Y	Y	Y	Y	Y	Y	N	Y	8
Boszczowski	Y	Y	Y	Y	Y	Y	Y	Y	Y	9
Cabanel	Y	Y	Y	Y	Y	Y	Y	Y	Y	9
Can	Y	Y	Y	Y	Y	Y	Y	Y	Y	9
Cannatelli	Y	Y	Y	Y	Y	Y	Y	N	Y	8
Cejas	Y	Y	Y	Y	Y	Y	Y	N	Y	8
Chen 2021	Y	Y	Y	Y	Y	Y	Y	N	Y	8
Chen 2022	N	Y	N	N	N	Y	Y	Y	Y	5
Cheng	Y	Y	Y	Y	Y	Y	Y	N	Y	8
Cheong	Y	Y	Y	N	N	Y	Y	N	Y	6
Cienfuegos_Gallet	Y	Y	Y	N	N	Y	Y	N	Y	6
Conceicão-Neto	Y	Y	Y	Y	Y	Y	Y	Y	Y	9
Di_Pilato	Y	Y	Y	Y	Y	Y	Y	N	Y	8
Di_Tella	Y	Y	Y	Y	Y	Y	Y	N	Y	8
Dong	Y	Y	Y	Y	Y	Y	Y	Y	Y	9
D'Onofrio	Y	Y	Y	Y	Y	Y	Y	N	Y	8
Elias	Y	Y	Y	Y	Y	Y	Y	N	Y	8
Esposito	Y	Y	Y	Y	Y	Y	Y	Y	Y	9
Foldes	Y	Y	Y	Y	Y	Y	Y	N	Y	8
Garcia-Fulgueiras	Y	Y	Y	Y	Y	Y	Y	N	Y	8
Garza-Ramos	Y	Y	Y	N	N	Y	Y	N	Y	6
Gentile	Y	Y	Y	Y	Y	Y	Y	N	Y	8
Haeili	Y	Y	Y	N	N	Y	Y	N	Y	6

Halaby	Y	Y	Y	Y	Y	Y	Y	N	Y	8
Hamel	Y	Y	Y	Y	Y	Y	Y	Y	Y	9
Hu	Y	Y	Y	Y	Y	Y	Y	N	Y	8
Huang	Y	Y	Y	N	N	Y	Y	Y	Y	7
Huang 2022	Y	Y	Y	Y	Y	Y	Y	Y	Y	9
Jaidane	Y	Y	Y	Y	Y	Y	Y	N	Y	8
Jayol 2016	Y	Y	Y	Y	Y	Y	Y	N	Y	8
Jayol 2018	Y	Y	Y	N	N	Y	Y	N	Y	6
Jin	Y	Y	Y	Y	Y	Y	Y	Y	Y	9
Karampatakis	Y	Y	Y	Y	Y	Y	Y	N	Y	8
Kaza	N	Y	N	Y	Y	Y	Y	N	Y	6
Khoshbayan	Y	Y	Y	Y	Y	Y	Y	N	Y	8
Kim	Y	Y	Y	N	N	Y	Y	N	Y	6
Kis	Y	Y	Y	Y	Y	Y	Y	Y	Y	9
Kong	Y	Y	Y	Y	Y	Y	Y	N	Y	8
Kumar	Y	Y	Y	Y	Y	Y	Y	N	Y	8
Lalaoui	Y	Y	Y	Y	Y	Y	Y	N	Y	8
Lee	Y	Y	Y	N	N	Y	Y	N	Y	6
Leung	Y	Y	Y	Y	Y	Y	Y	N	Y	8
Liu	Y	Y	Y	Y	Y	Y	Y	Y	Y	9
Lomonaco	Y	Y	Y	N	N	N	Y	N	Y	5
Longo	Y	Y	Y	Y	Y	Y	Y	N	Y	8
Lopez_Camacho	N	Y	N	Y	Y	Y	Y	Y	Y	7
Macesic	Y	Y	Y	N	N	Y	Y	Y	Y	7
Malli	Y	Y	Y	Y	Y	Y	Y	Y	Y	9
Mansour	Y	Y	Y	N	N	Y	Y	N	Y	6
Mathur	Y	Y	Y	Y	N	N	Y	Y	Y	7
Mavroidi	Y	Y	Y	Y	Y	Y	Y	Y	Y	9
Mavroidi 2019	Y	Y	Y	Y	Y	Y	Y	N	Y	8
Mills	Y	Y	Y	Y	Y	Y	Y	Y	Y	9
Mirshekar	Y	Y	Y	Y	Y	Y	Y	Y	Y	9
Moghimi	N	Y	N	Y	N	Y	Y	Y	Y	6
Naha	N	Y	N	Y	Y	Y	Y	Y	Y	7
Nawfal_Dagher	Y	Y	Y	Y	Y	Y	Y	N	Y	8
Ngbede	Y	Y	Y	Y	Y	Y	Y	N	Y	8
Nguyen	Y	Y	Y	Y	Y	Y	Y	Y	Y	9
Niazadeh	Y	Y	Y	Y	Y	Y	Y	Y	Y	9
Nirwan	Y	Y	Y	Y	Y	Y	Y	Y	Y	9
Nordmann	N	Y	N	Y	Y	Y	Y	Y	Y	7
Novovic	Y	Y	Y	Y	Y	Y	Y	N	Y	8

Okdah	Y	Y	Y	Y	Y	Y	Y	Y	Y	9
Olaitan	Y	Y	Y	Y	Y	Y	Y	Y	Y	9
Otter	Y	Y	Y	Y	Y	Y	Y	N	Y	8
Palani	Y	Y	Y	Y	Y	Y	Y	N	Y	8
Palmieri	Y	Y	Y	Y	Y	Y	Y	Y	Y	9
Pitt	Y	Y	Y	Y	Y	Y	Y	N	Y	8
Poirel	Y	Y	Y	N	N	Y	Y	N	Y	8
Popa	Y	Y	Y	Y	Y	Y	Y	Y	Y	9
Pragasam	Y	Y	Y	Y	Y	Y	Y	N	Y	8
Pu	Y	Y	Y	Y	Y	Y	Y	Y	Y	9
Rimoldi	Y	Y	Y	Y	Y	Y	Y	N	Y	8
Roch	Y	Y	Y	Y	Y	Y	Y	N	Y	8
Rocha	Y	Y	Y	Y	Y	Y	Y	N	Y	8
Rocha	Y	Y	Y	Y	Y	Y	Y	Y	Y	9
Rubic	Y	Y	Y	Y	Y	Y	Y	Y	Y	9
Shamina	N	Y	N	Y	N	Y	Y	Y	Y	6
Shankar	Y	Y	Y	Y	Y	Y	Y	N	Y	8
Sharahi	Y	Y	Y	Y	Y	Y	Y	Y	Y	9
Singh	Y	Y	Y	Y	Y	Y	Y	N	Y	8
Sisti	Y	Y	Y	Y	Y	Y	Y	N	Y	8
Snyman	Y	Y	Y	Y	Y	Y	Y	Y	Y	9
Solgi	Y	Y	Y	Y	Y	Y	Y	Y	Y	9
Sonnevend	Y	Y	Y	Y	Y	Y	Y	N	Y	8
Tietgen	Y	Y	Y	N	N	Y	Y	N	Y	6
Torres	Y	Y	Y	N	N	Y	Y	N	Y	6
Uz_Zaman	Y	Y	Y	Y	Y	Y	Y	N	Y	8
Vendrik	Y	Y	Y	Y	Y	Y	Y	Y	Y	9
Wang	Y	Y	Y	Y	Y	Y	Y	Y	Y	9
Wright	Y	Y	Y	Y	Y	Y	Y	Y	Y	9
Xiao	Y	Y	Y	Y	Y	Y	Y	Y	Y	9
Xie	N	N	N	Y	Y	Y	Y	Y	Y	6
Yang	Y	Y	Y	Y	Y	Y	Y	Y	Y	9
Yap	N	N	N	Y	Y	Y	Y	N	Y	5
Yoshino	Y	Y	Y	Y	Y	Y	Y	N	Y	8
Yousfi	N	Y	N	Y	Y	Y	Y	N	Y	6
Zafer	Y	Y	Y	Y	Y	Y	Y	N	Y	8
Zhang	Y	Y	Y	Y	Y	Y	Y	N	Y	8
Zhu	Y	Y	Y	Y	Y	Y	Y	N	Y	9
