# Supplementary material for: Global prevalence of mutation in the mgrB gene among clinical isolates of colistin-resistant Klebsiella pneumoniae: a systematic review and meta-analysis
Source: Front Microbiol. 2024 Jun 7;15:1386478. doi: 10.3389/fmicb.2024.1386478 (PMC11190090; doi:10.3389/fmicb.2024.1386478)
Supplement: Supplementary file 3 [file Data_Sheet_2.PDF]

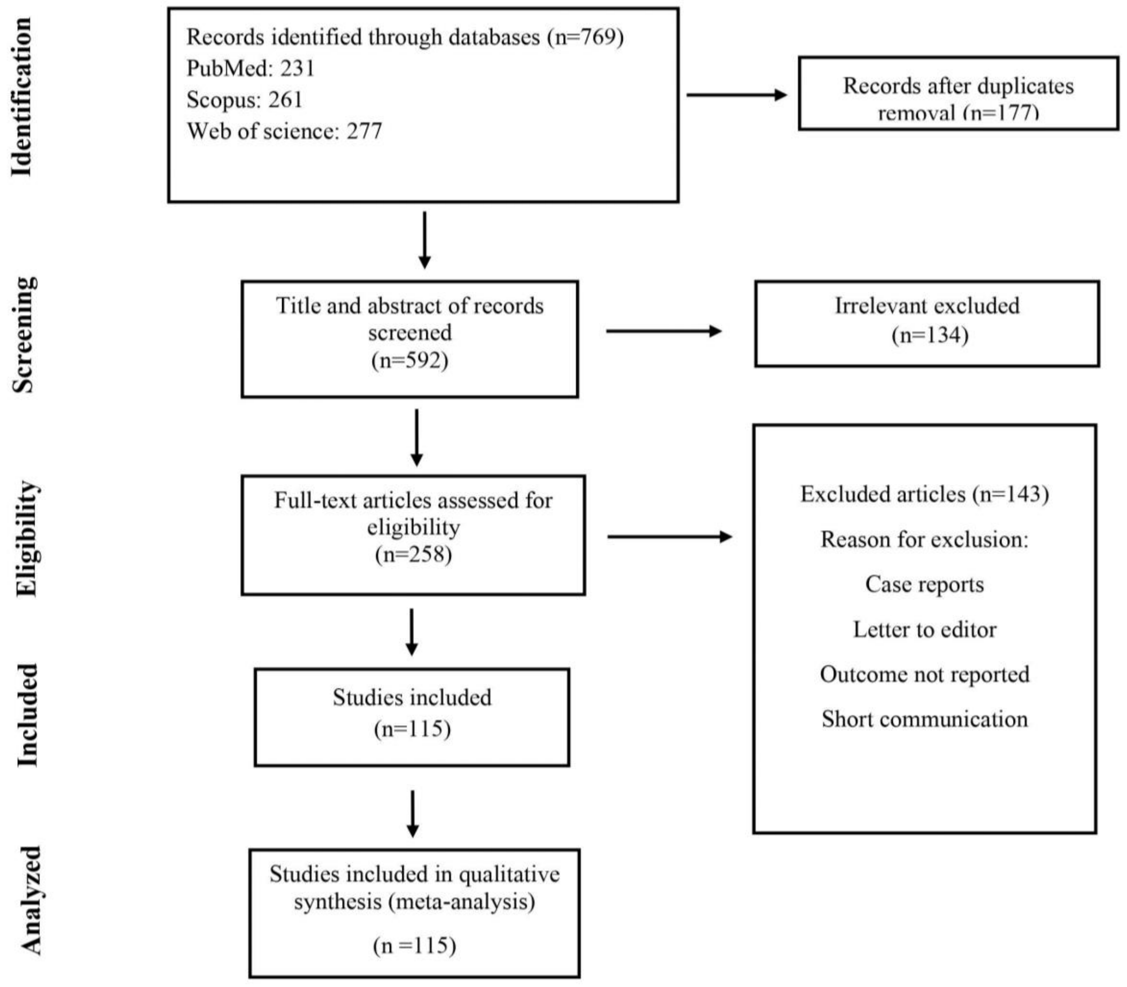

Figure 1: Flow chart of publication selection and their inclusion in the systematic review.

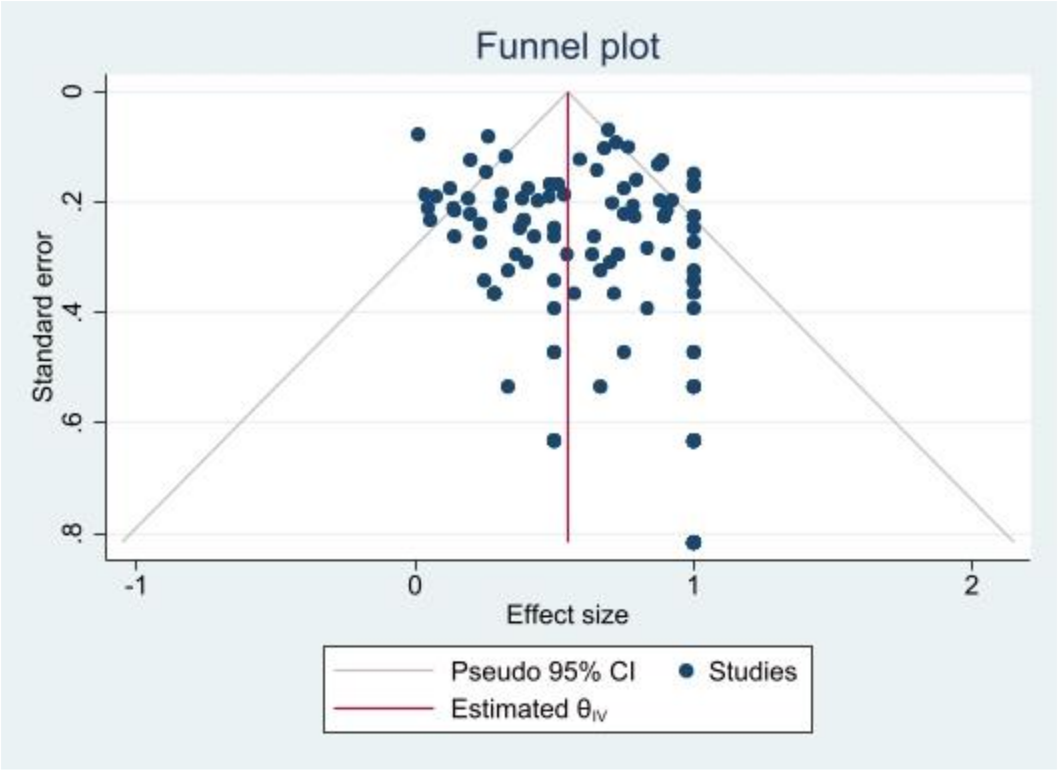

Figure 2: Funnel plot for meta-analysis.

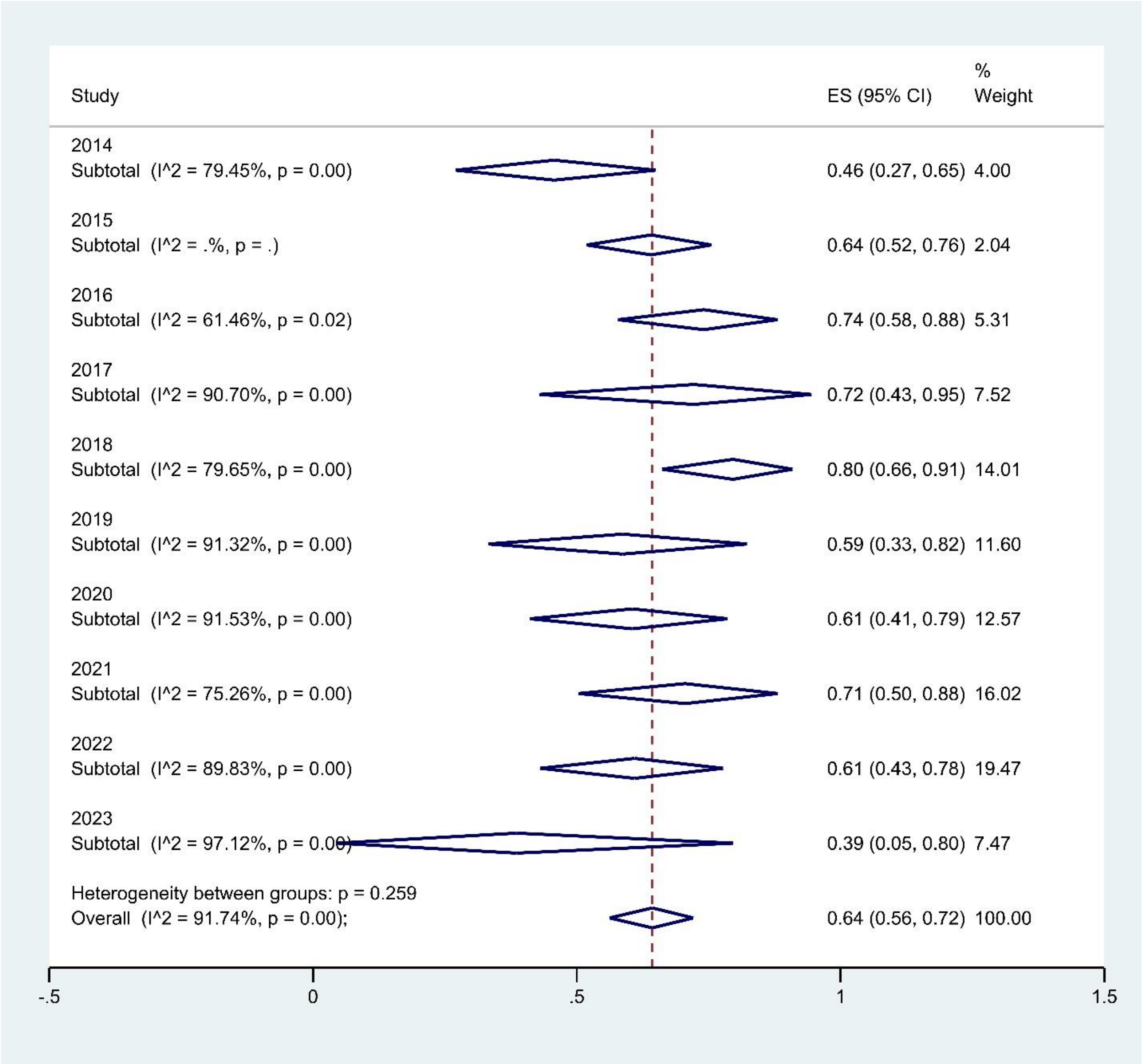

Figure 3: Subgroup meta-analysis for publishing year.

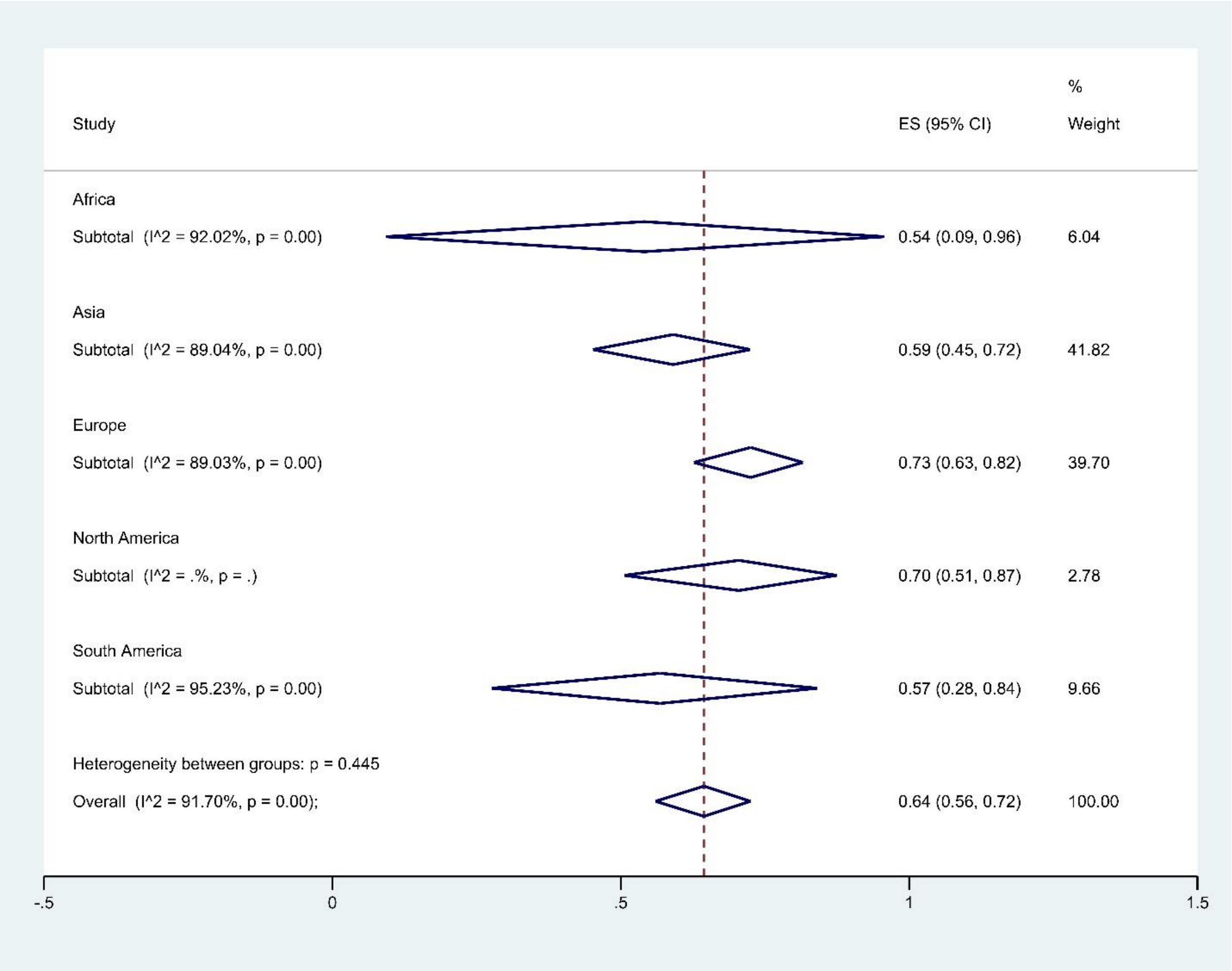

Figure 4: Subgroup meta-analysis for continents.

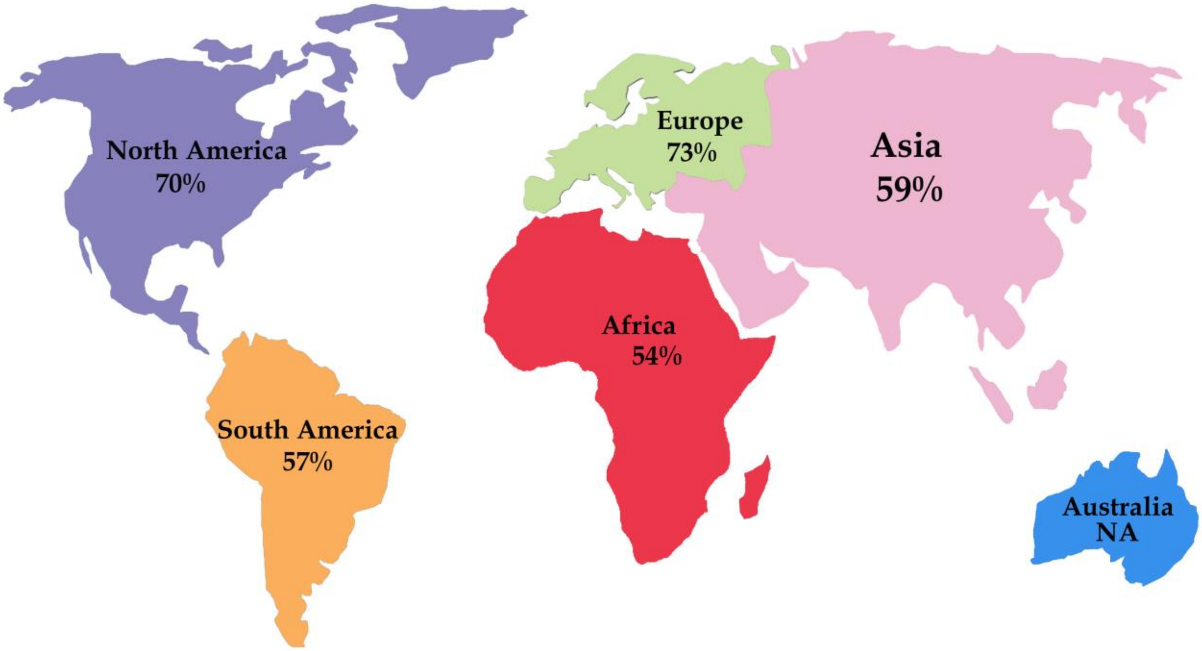

Figure 5: Distribution of *mgrB* mutated colistin-resistant *Klebsiella pneumoniae* isolates in each continent.

NA: not applicable.

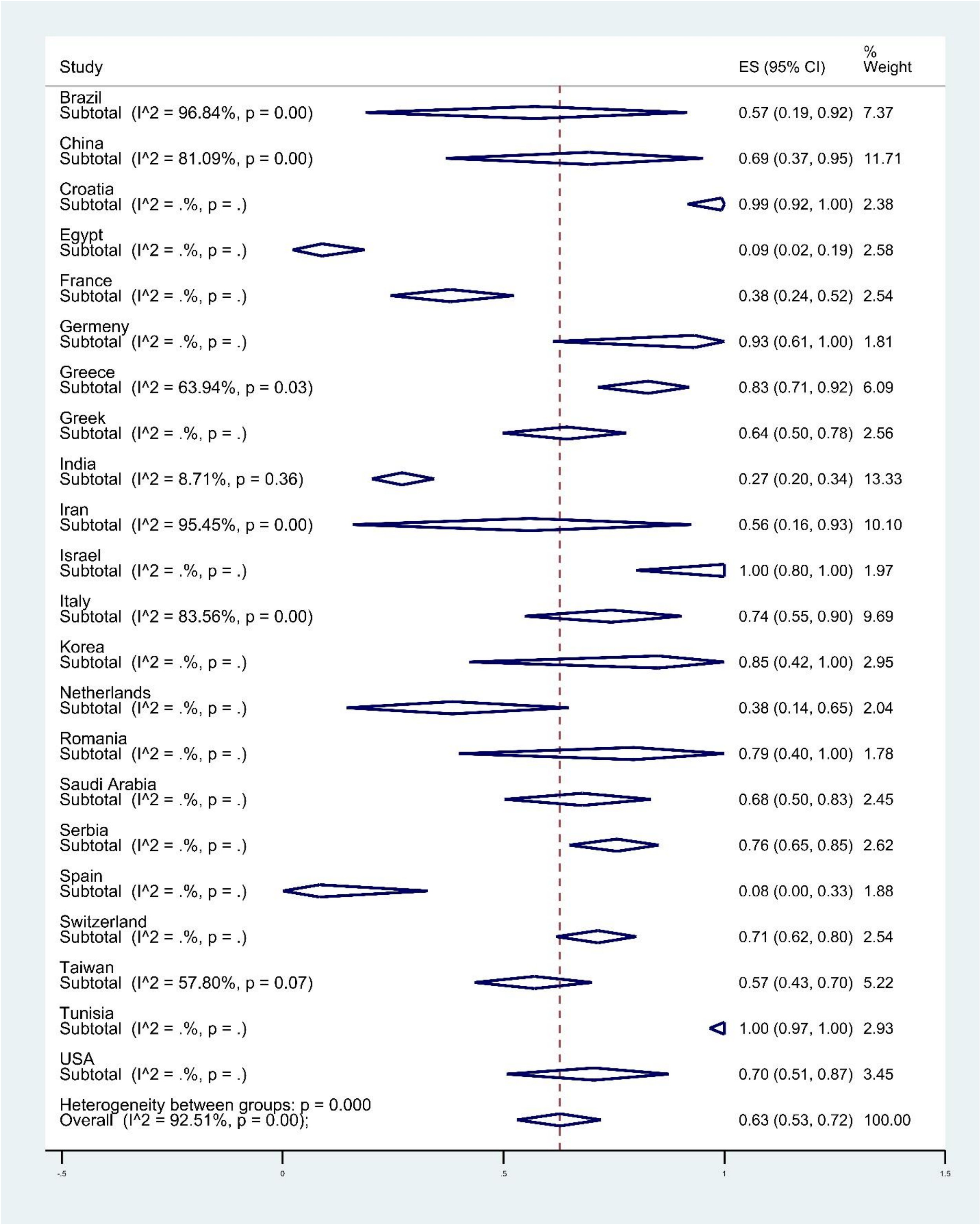

Figure 6: Subgroup meta-analysis for countries.

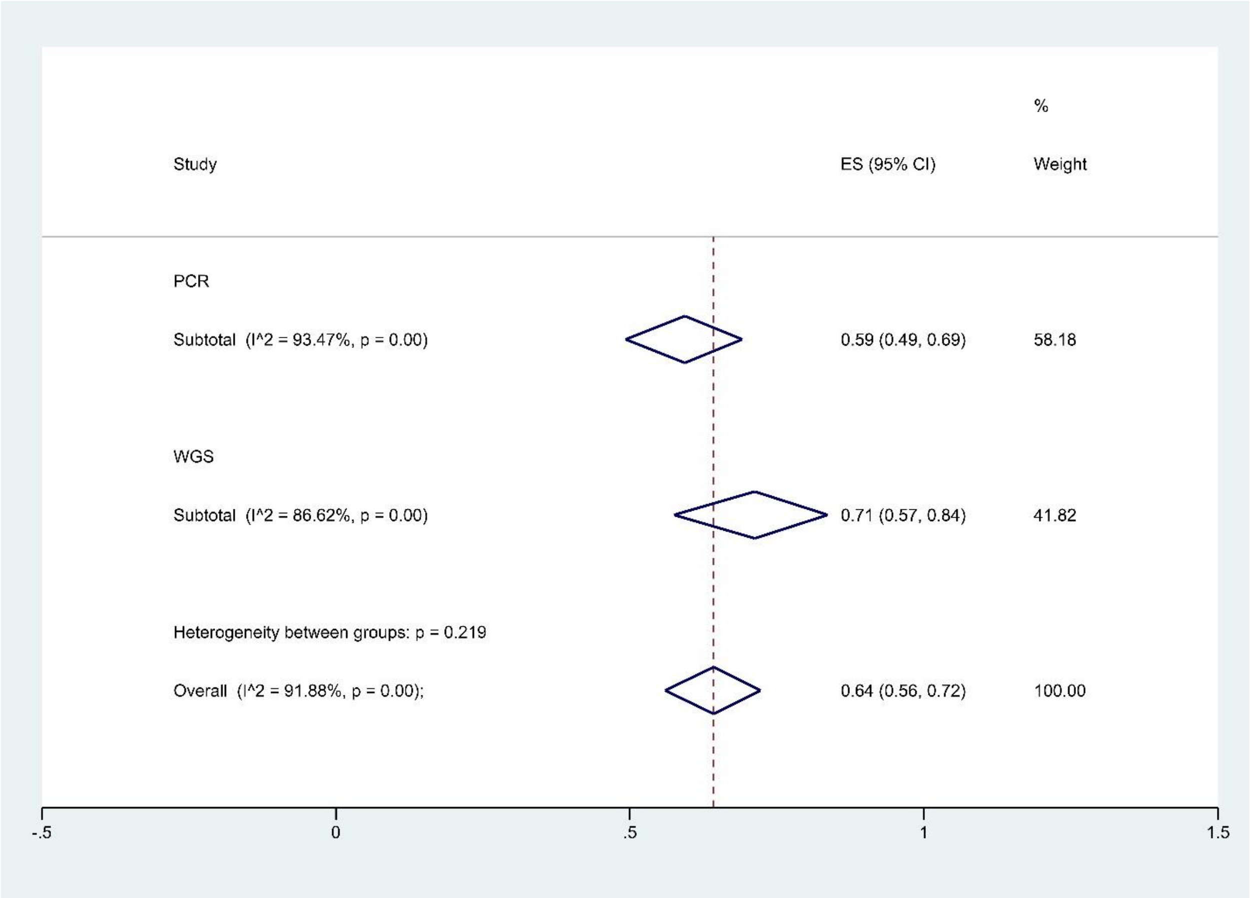

Figure 7: Subgroup meta-analysis for *mgrB* detection.

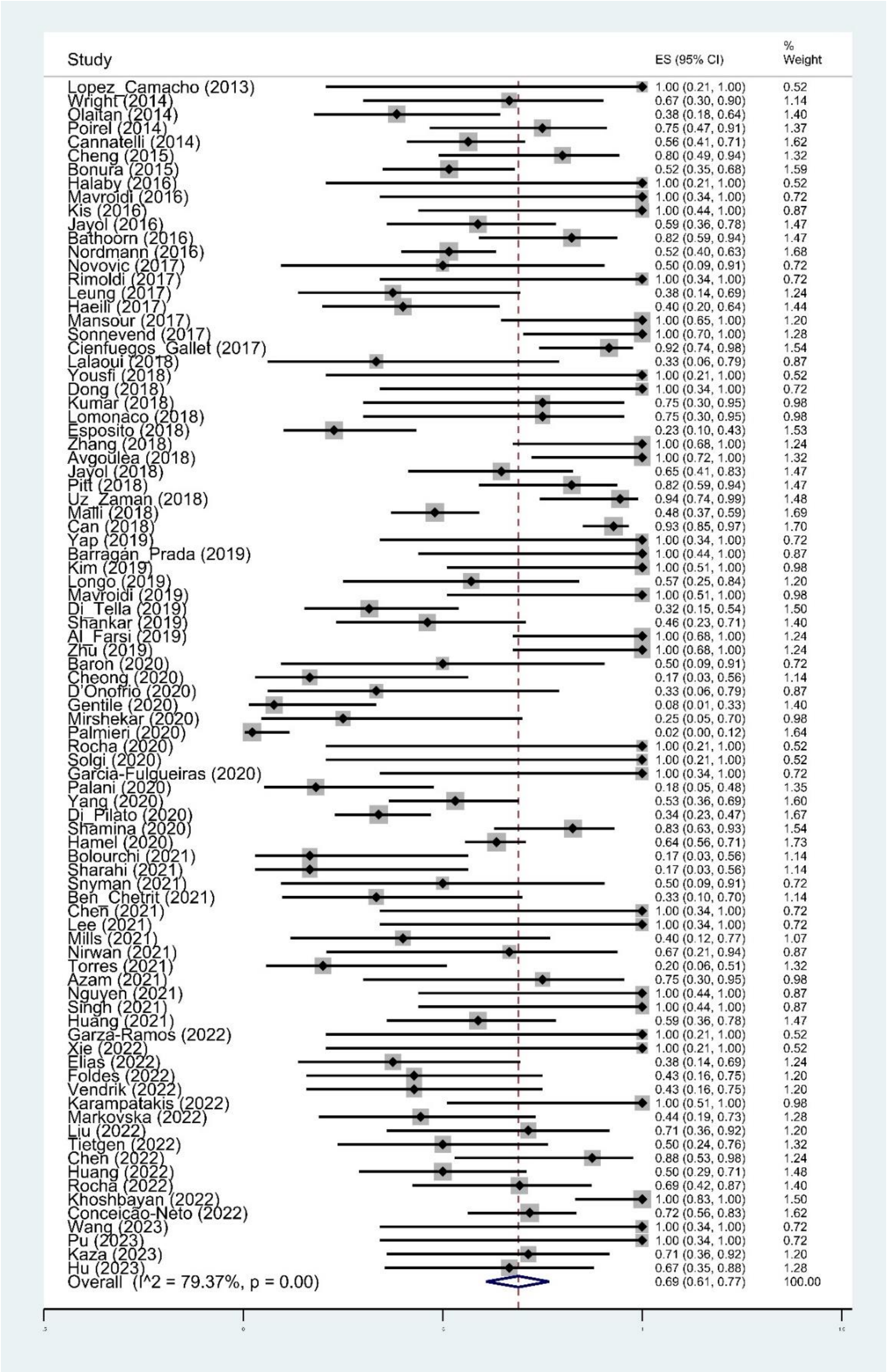

Figure 8: Forest plot for prevalence of mutation by insertional inactivation.

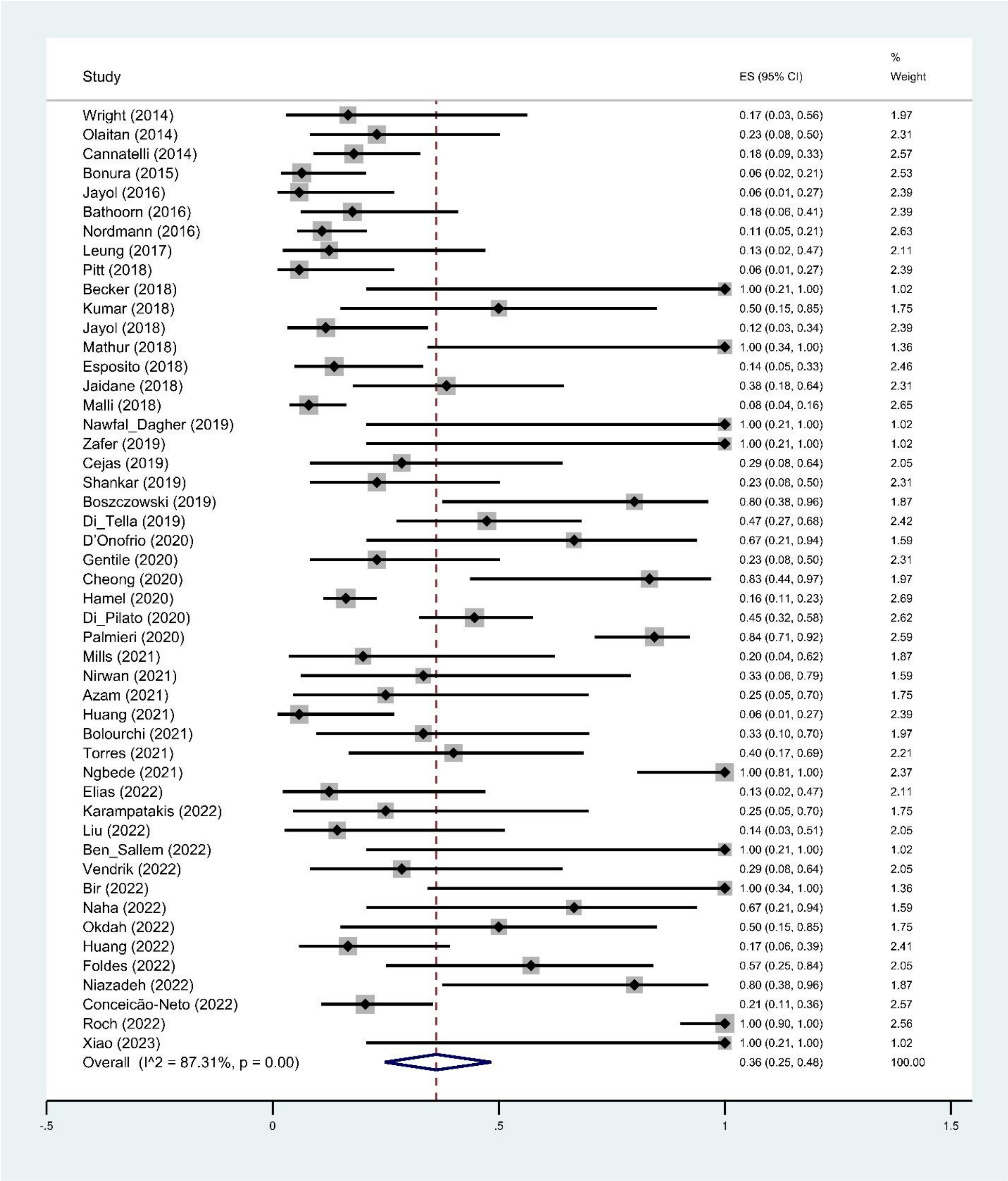

Figure 9: Forest plot for prevalence of mutation by substitution.

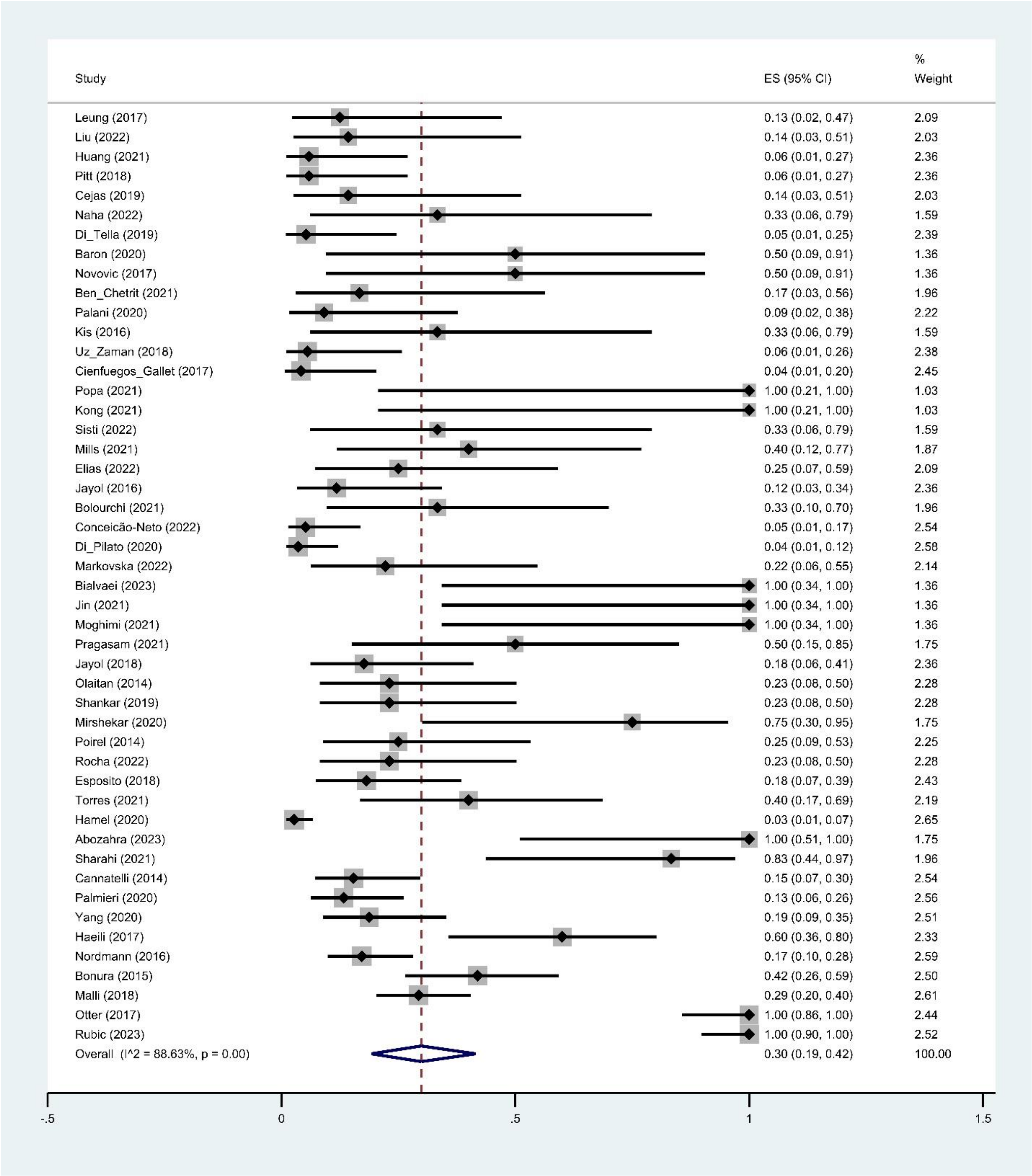

Figure 10: Forest plot for prevalence of nonsense mutation.

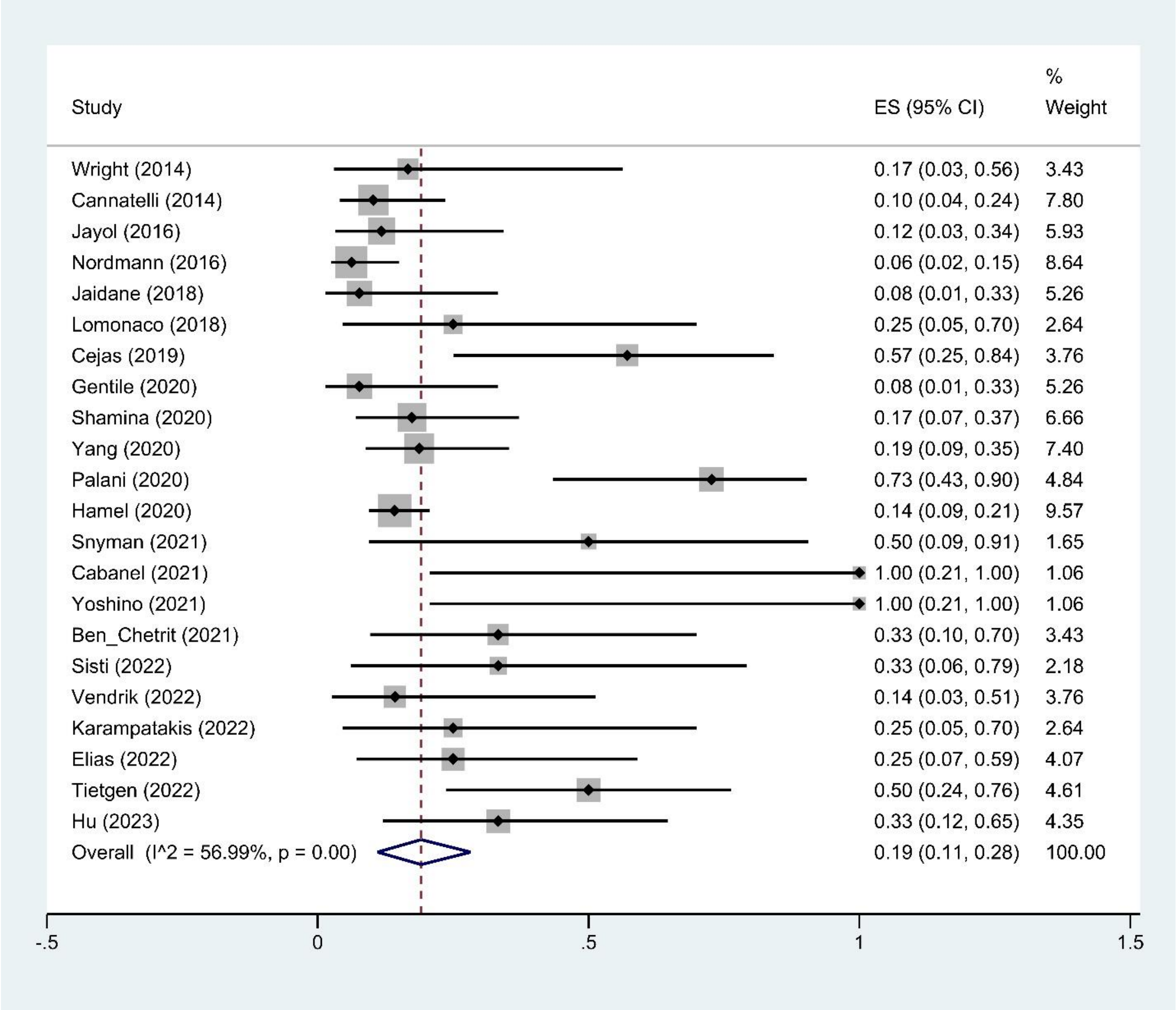

Figure 11: Forest plot for prevalence of complete deletion.

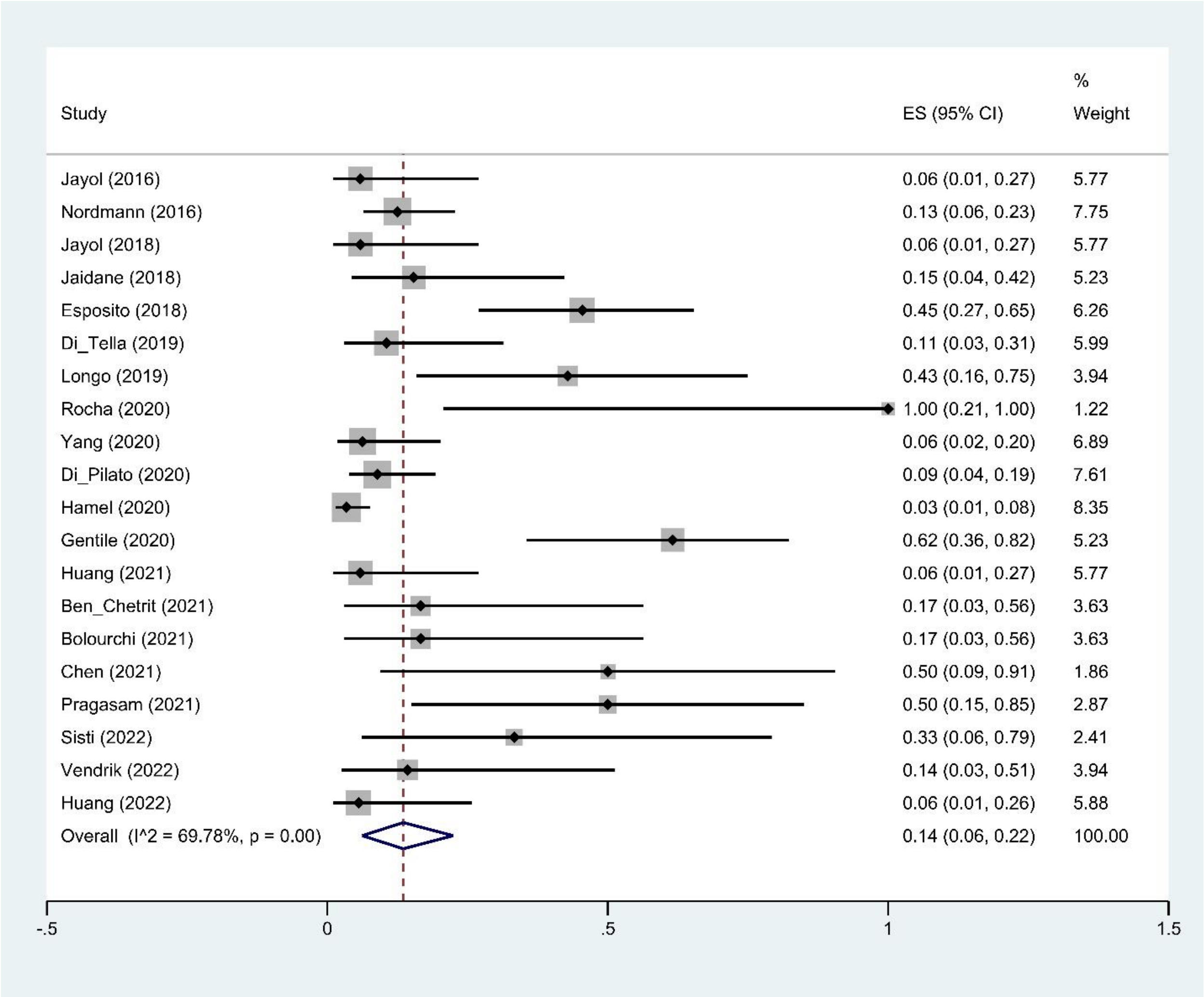

Figure 12: Forest plot for prevalence of partial deletion.
