# Supplementary figures and images for: Global prevalence of mutation in the mgrB gene among clinical isolates of colistin-resistant Klebsiella pneumoniae: a systematic review and meta-analysis
Source: Front Microbiol. 2024 Jun 7;15:1386478. doi: 10.3389/fmicb.2024.1386478 (PMC11190090; doi:10.3389/fmicb.2024.1386478)

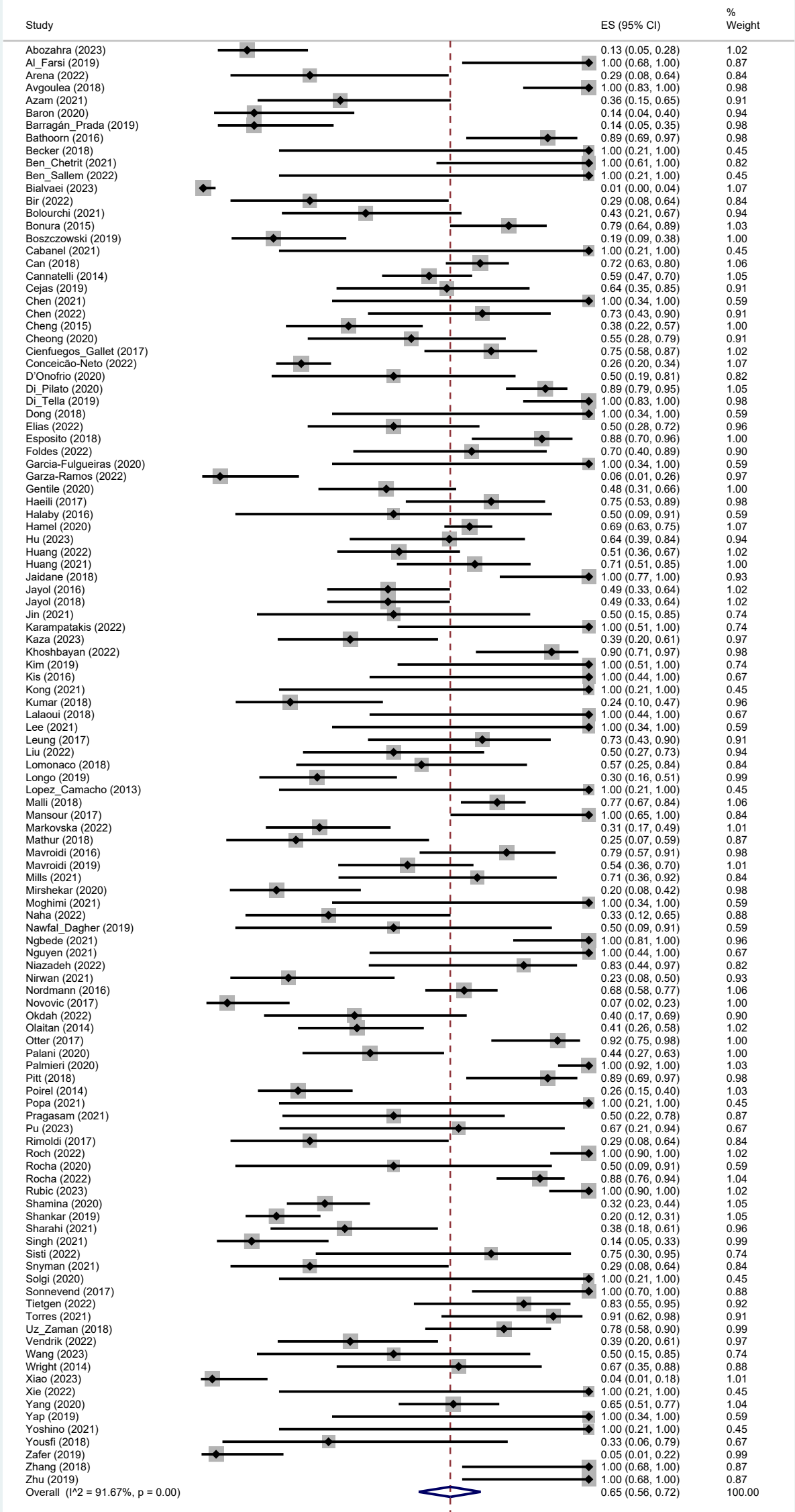

Supplement: Supplementary file 4 [file Data_Sheet_3.PDF]
