# Supplementary material for: Global prevalence of mutation in the mgrB gene among clinical isolates of colistin-resistant Klebsiella pneumoniae: a systematic review and meta-analysis
Source: Front Microbiol. 2024 Jun 7;15:1386478. doi: 10.3389/fmicb.2024.1386478 (PMC11190090; doi:10.3389/fmicb.2024.1386478)
Supplement: Supplementary file 5 [file Data_Sheet_4.PDF]

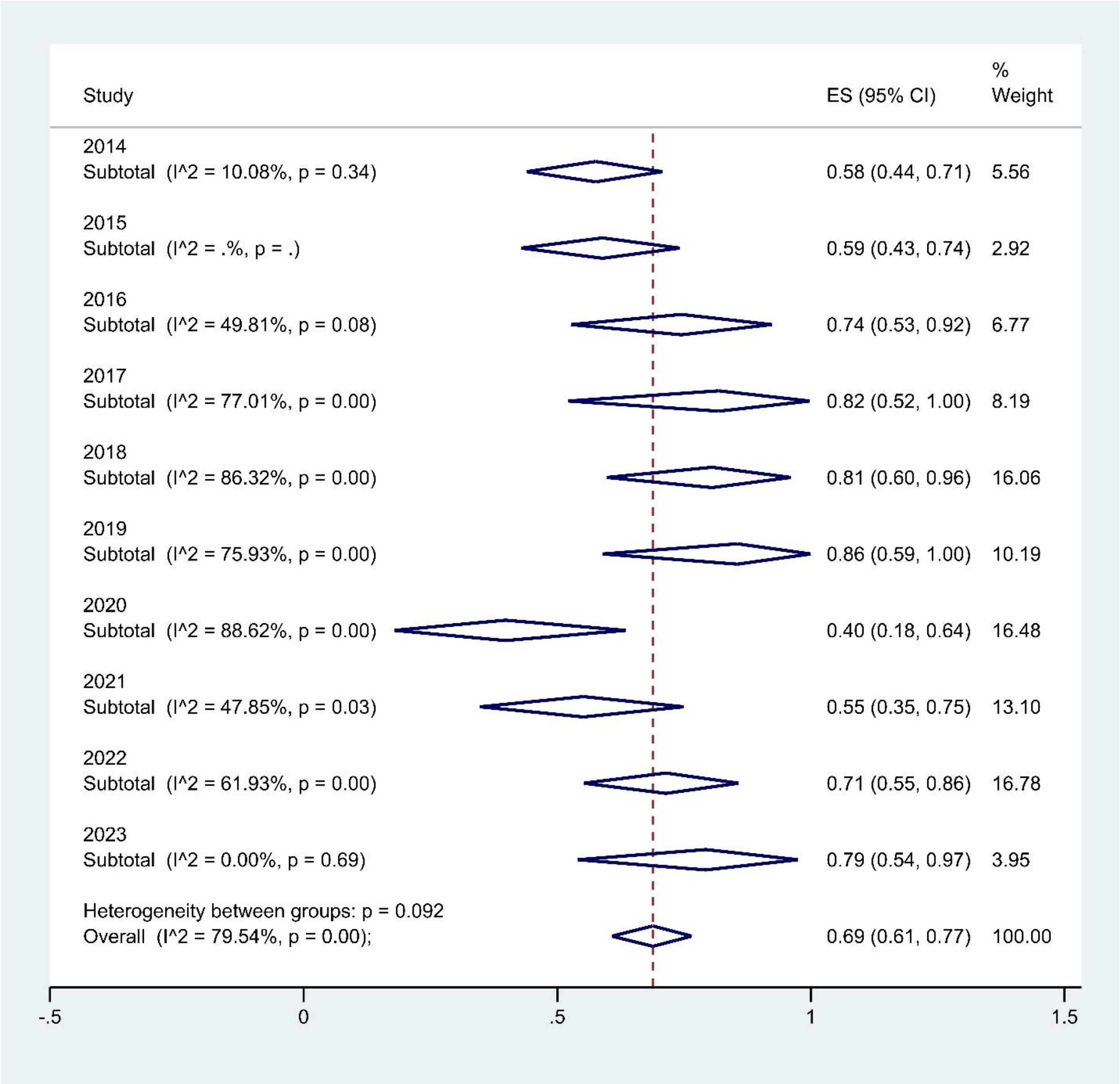

Figure 1: Subgroup meta-analysis for the year of mutation by insertional inactivation.

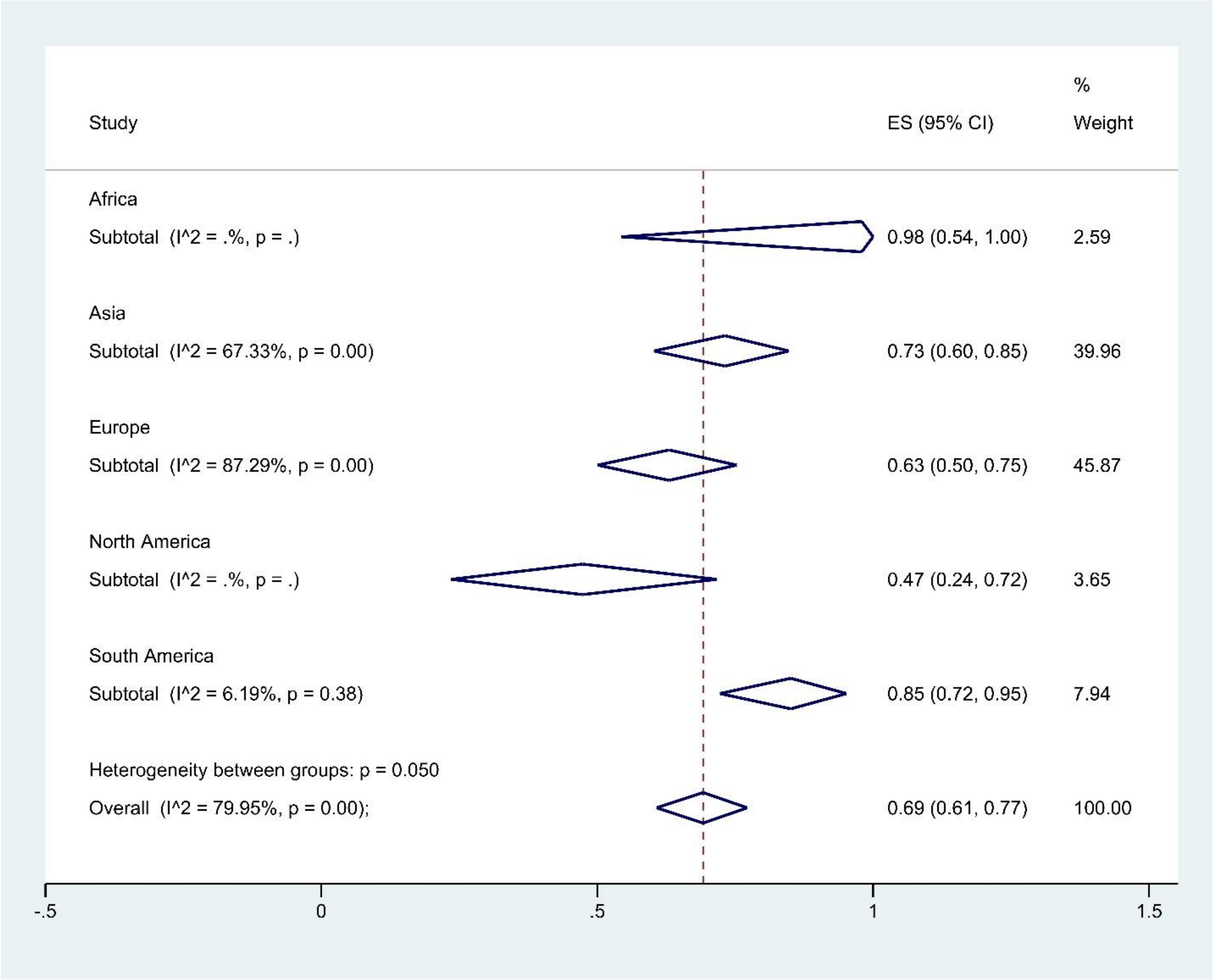

Figure 2: Subgroup meta-analysis for the continent of mutation by insertional inactivation.

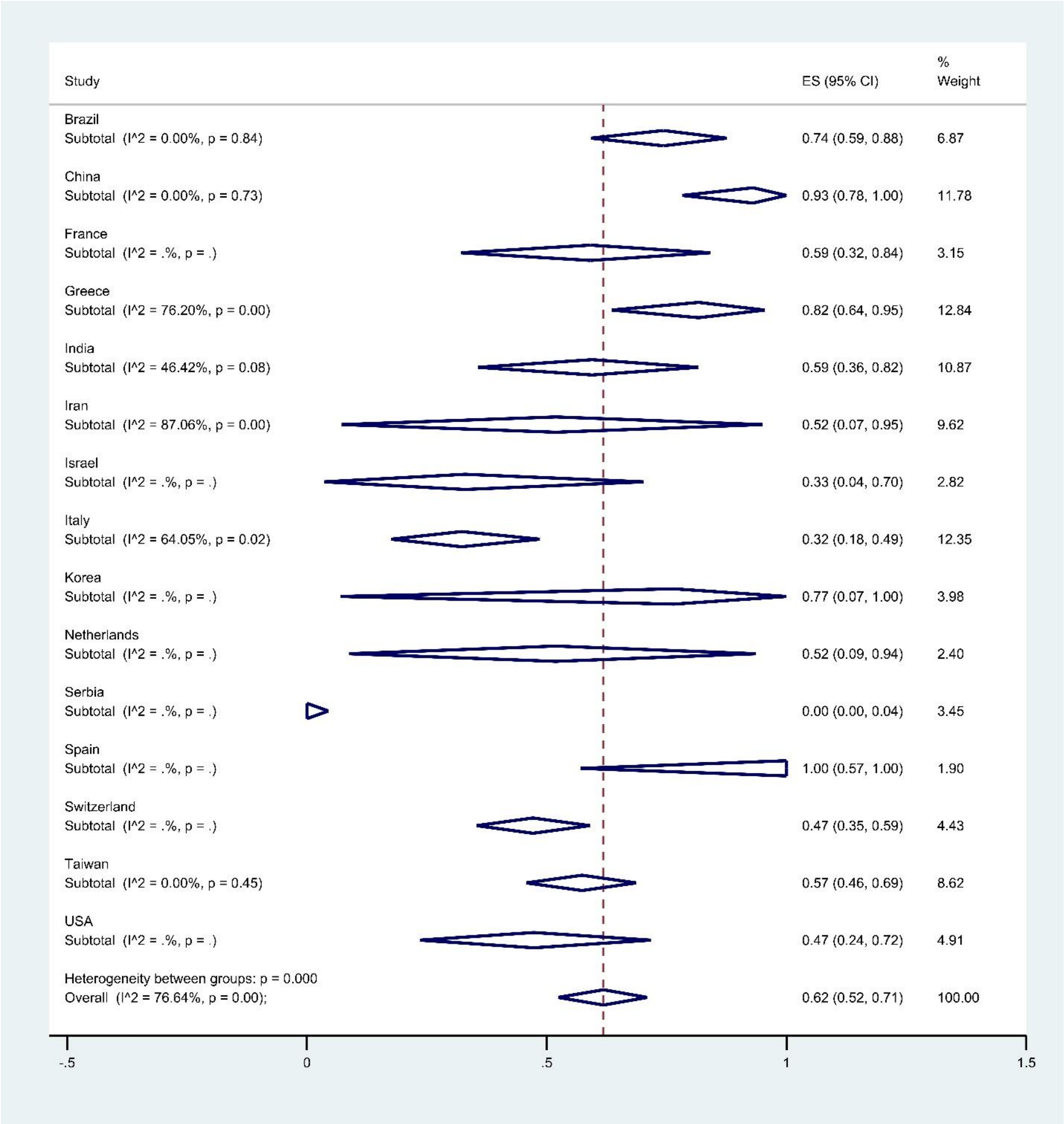

Figure 3: Subgroup meta-analysis for country of mutation by insertional inactivation.

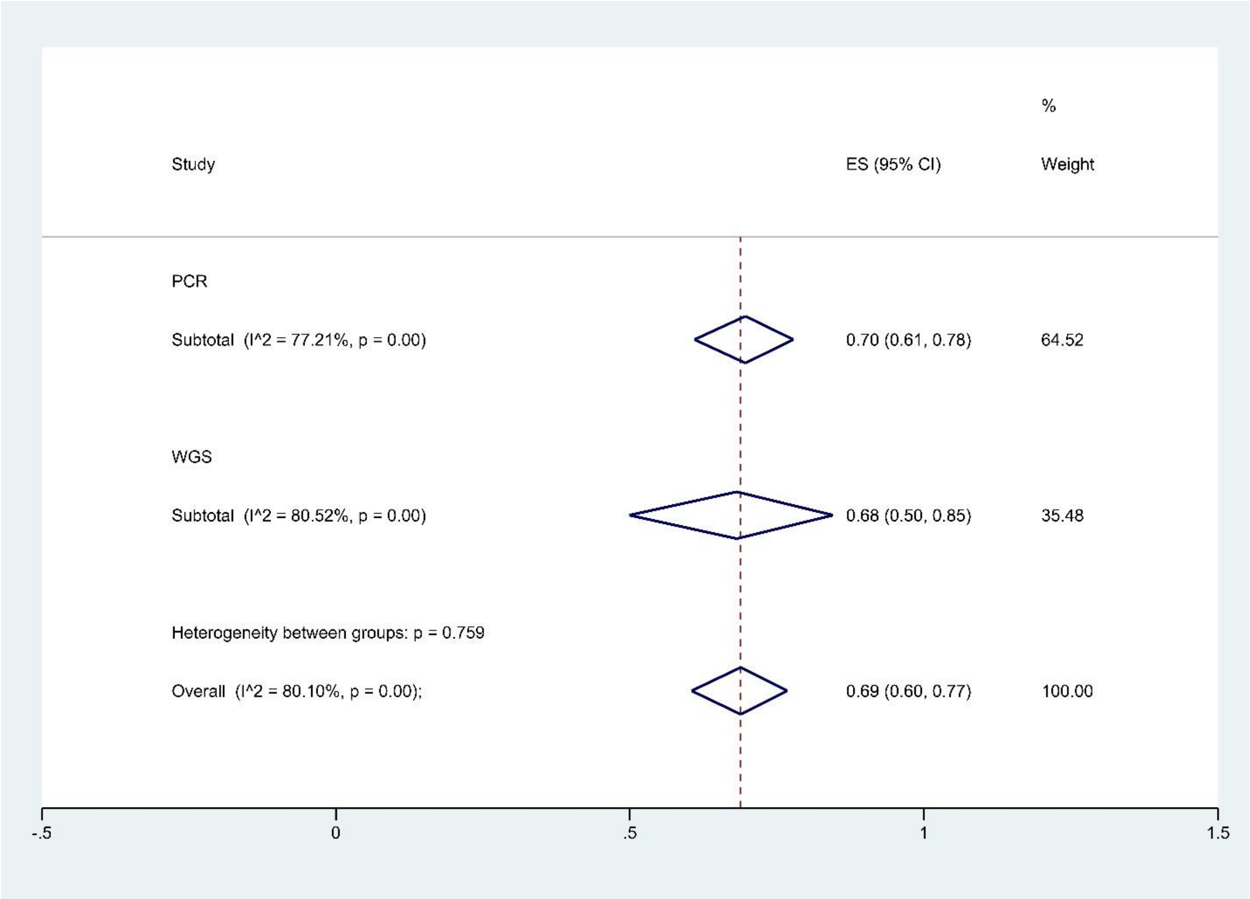

Figure 4: Subgroup meta-analysis for the method of mutation by insertional inactivation.

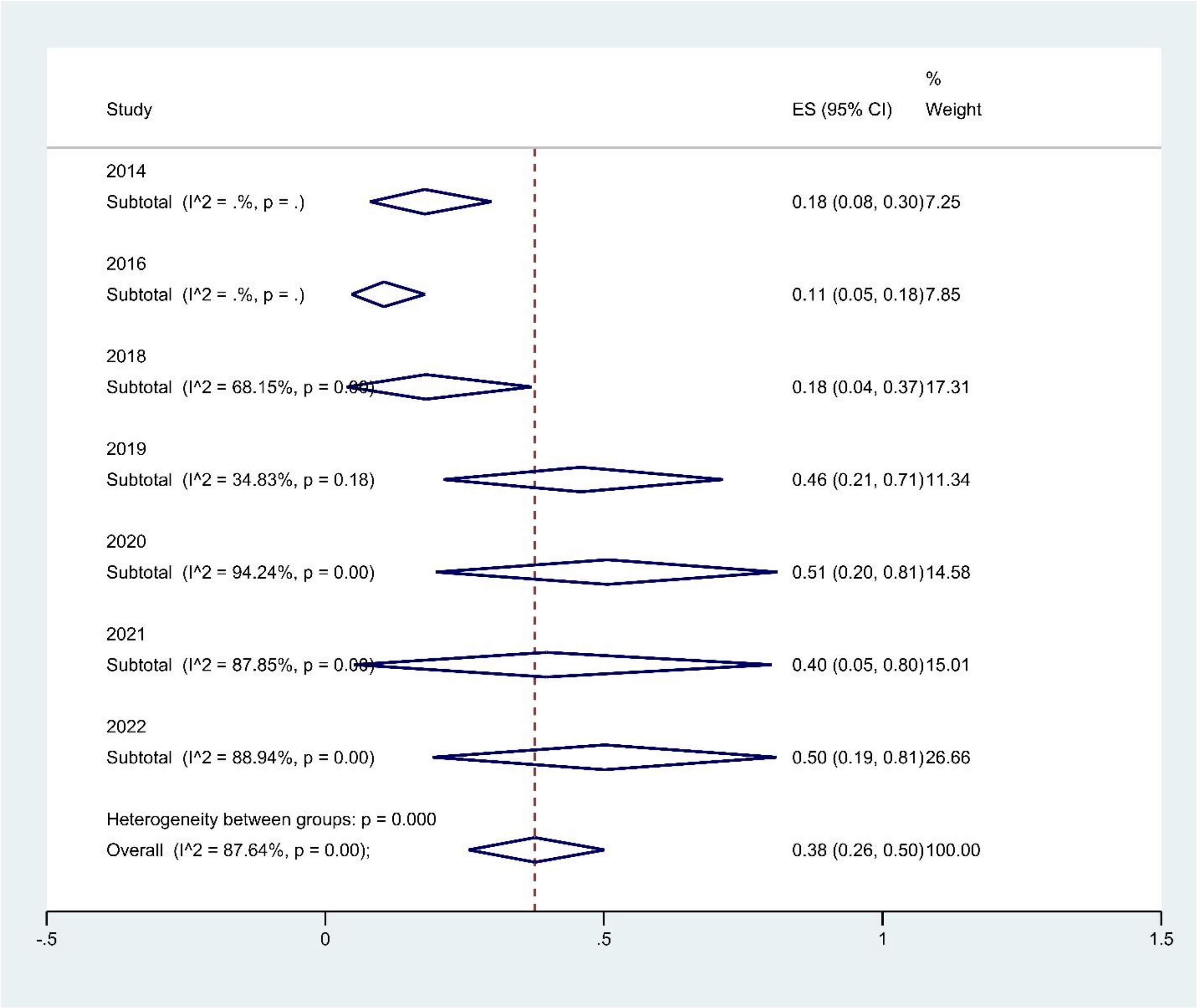

Figure 5: Subgroup meta-analysis for the year of substitution mutation.

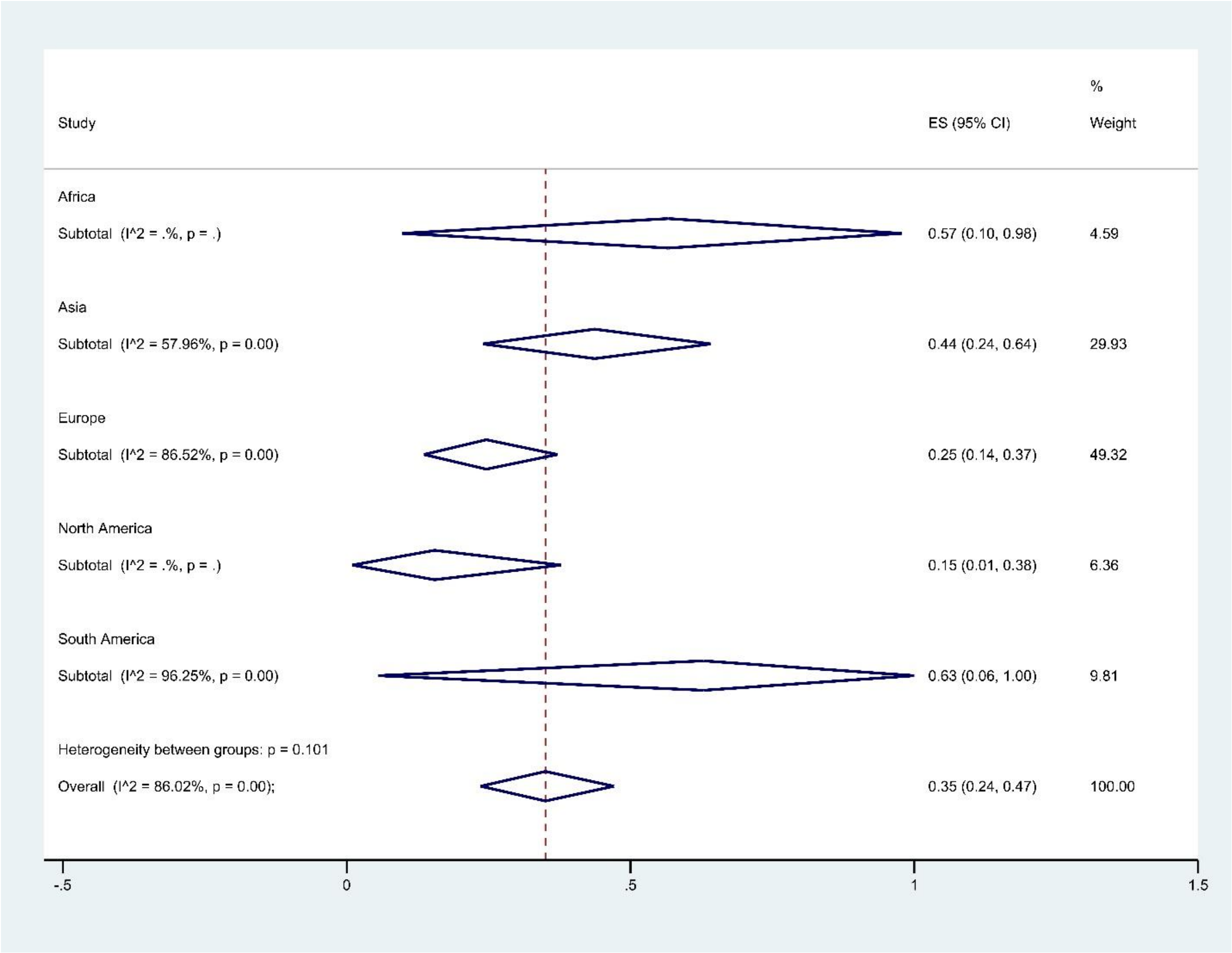

Figure 6: Subgroup meta-analysis for the continent of substitution mutation.

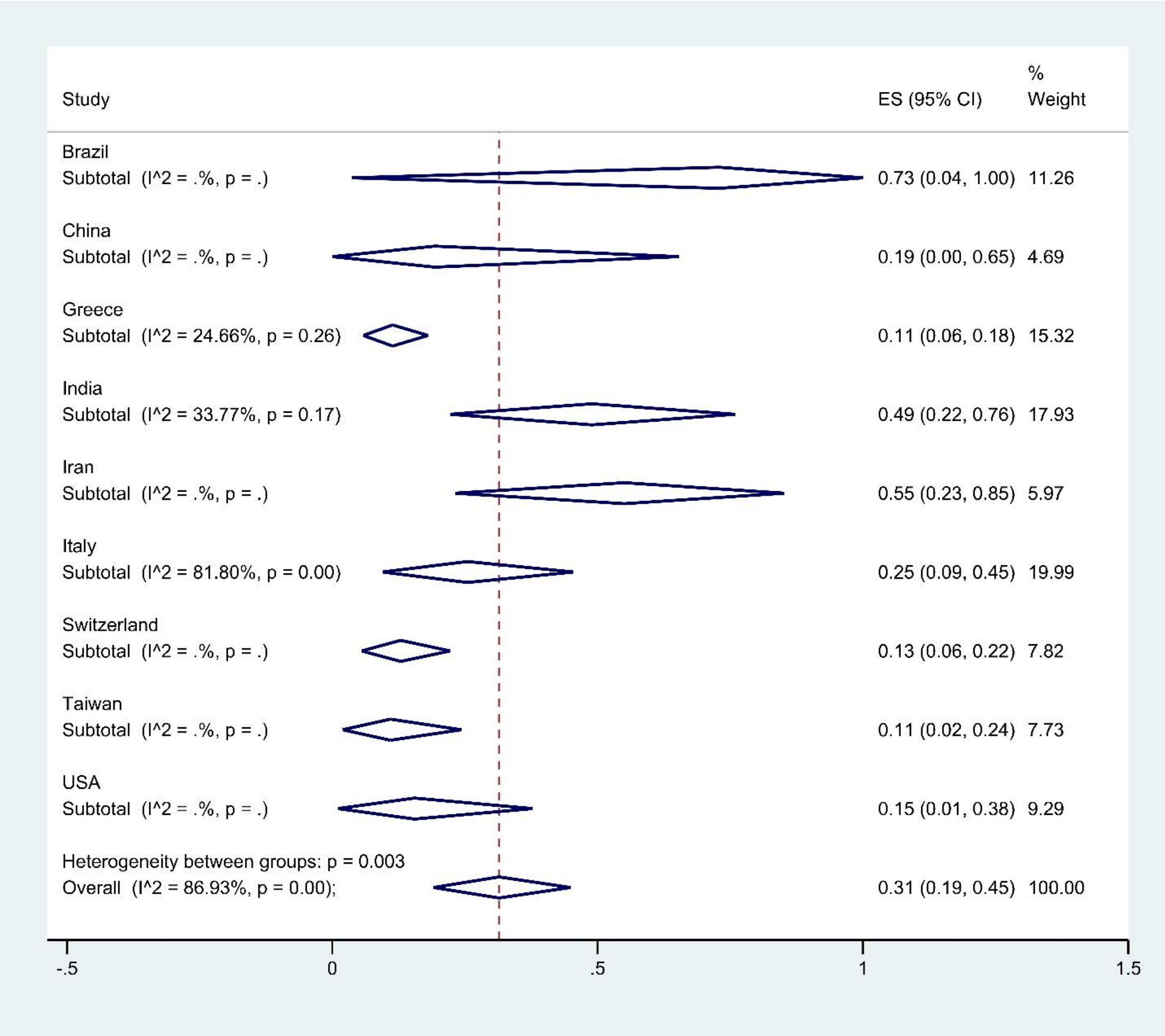

Figure 7: Subgroup meta-analysis for country of substitution mutation.

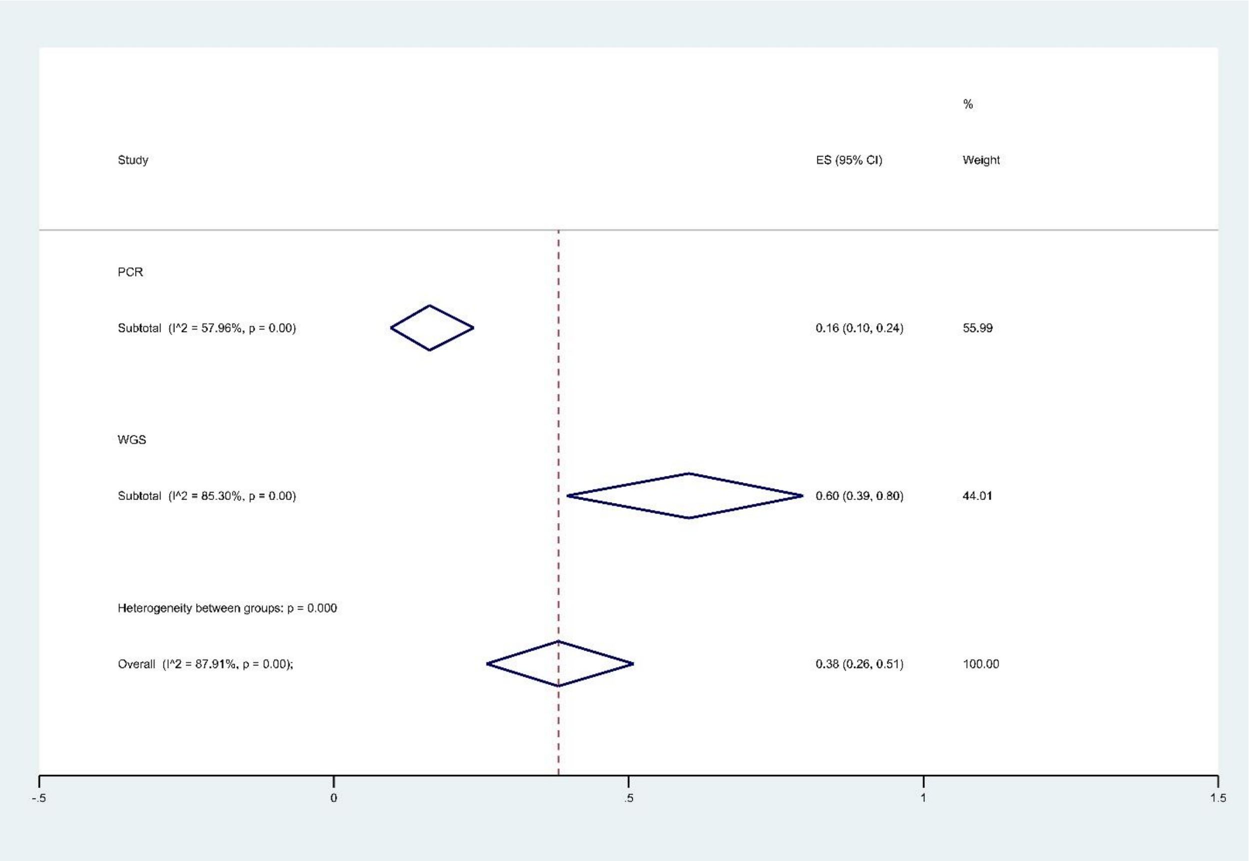

Figure 8: Subgroup meta-analysis for the method of substitution mutation.

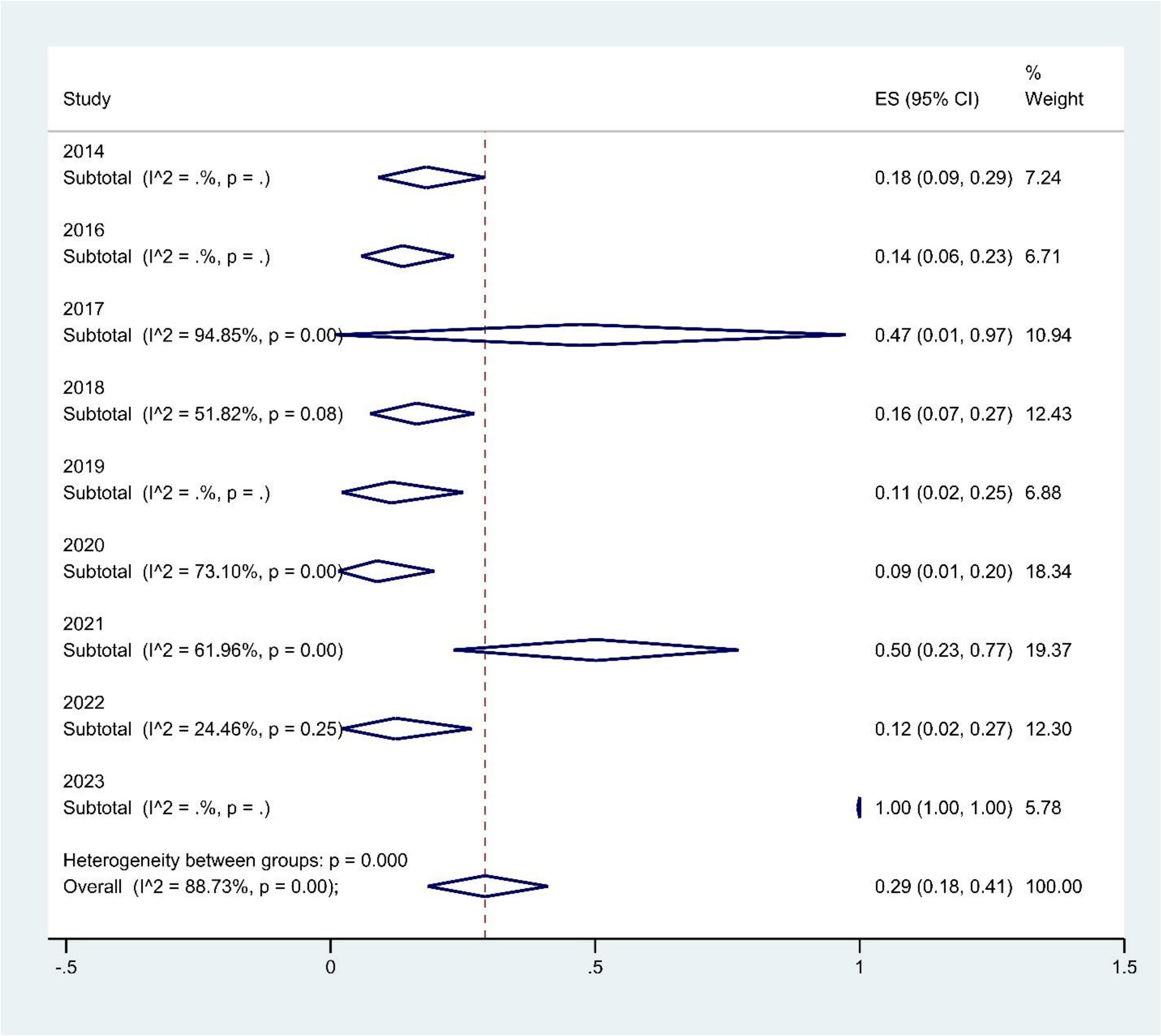

Figure 9: Subgroup meta-analysis for the year of nonsense mutation.

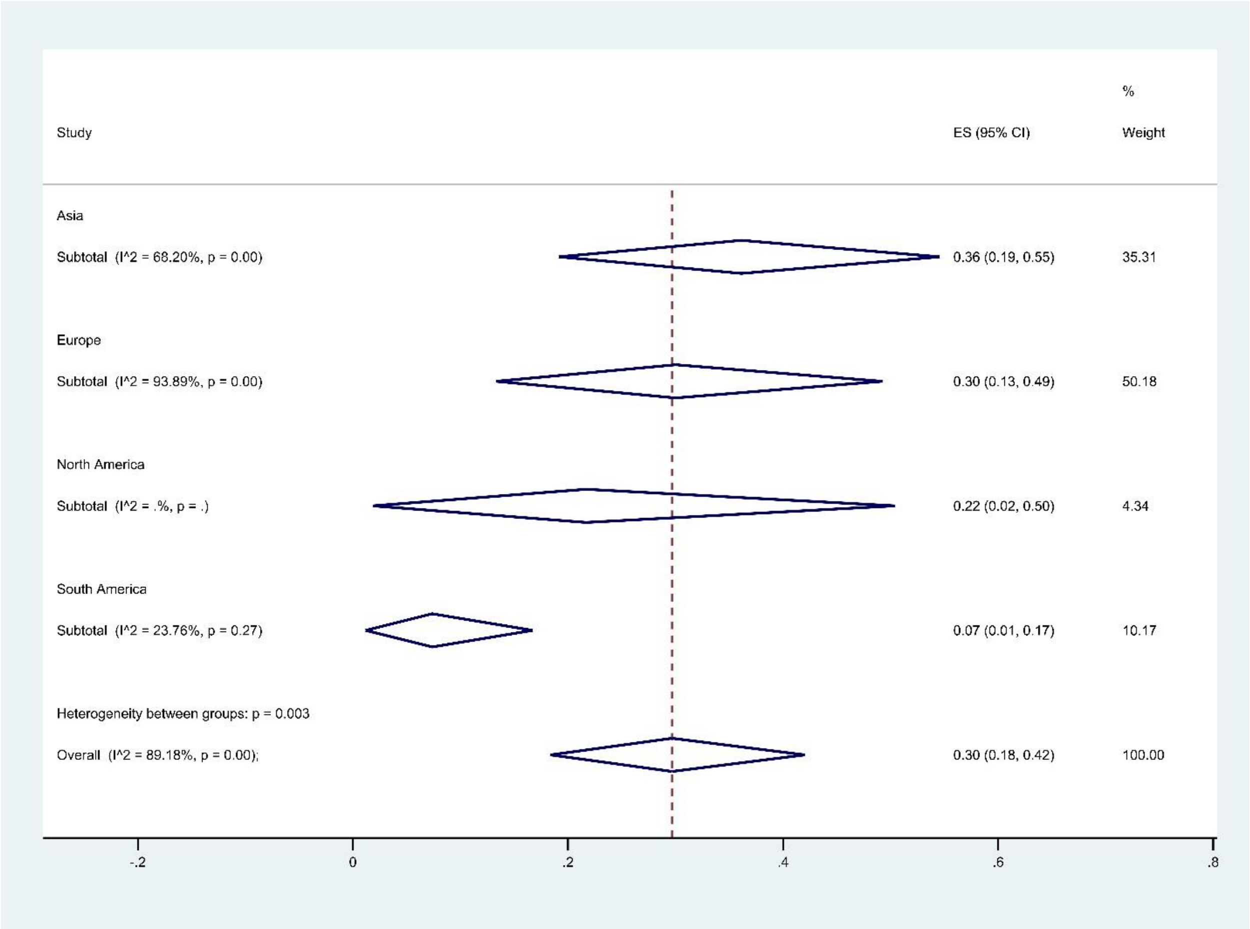

Figure 10: Subgroup meta-analysis for the continent of nonsense mutation.

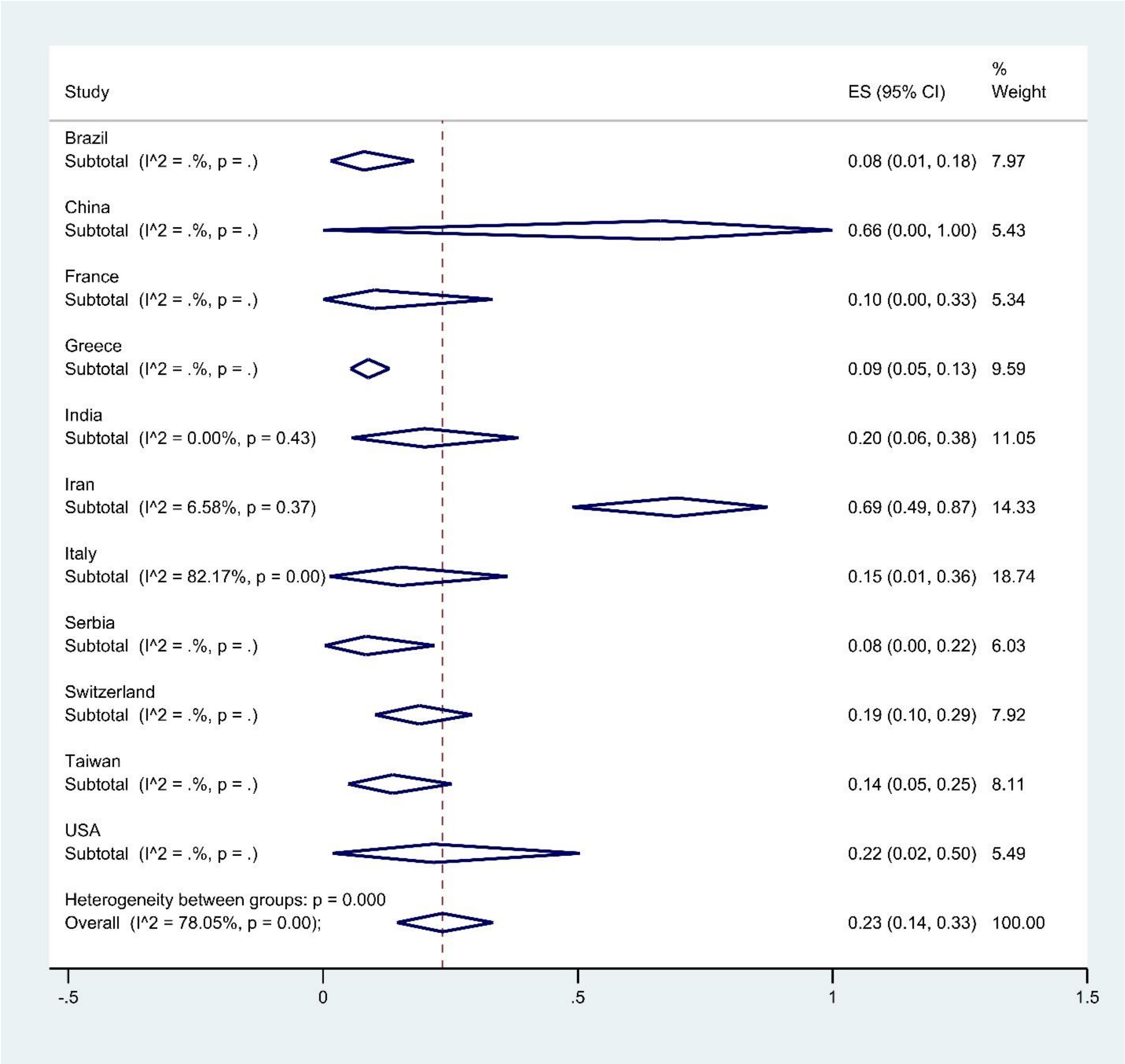

Figure 11: Subgroup meta-analysis for country of nonsense mutation.

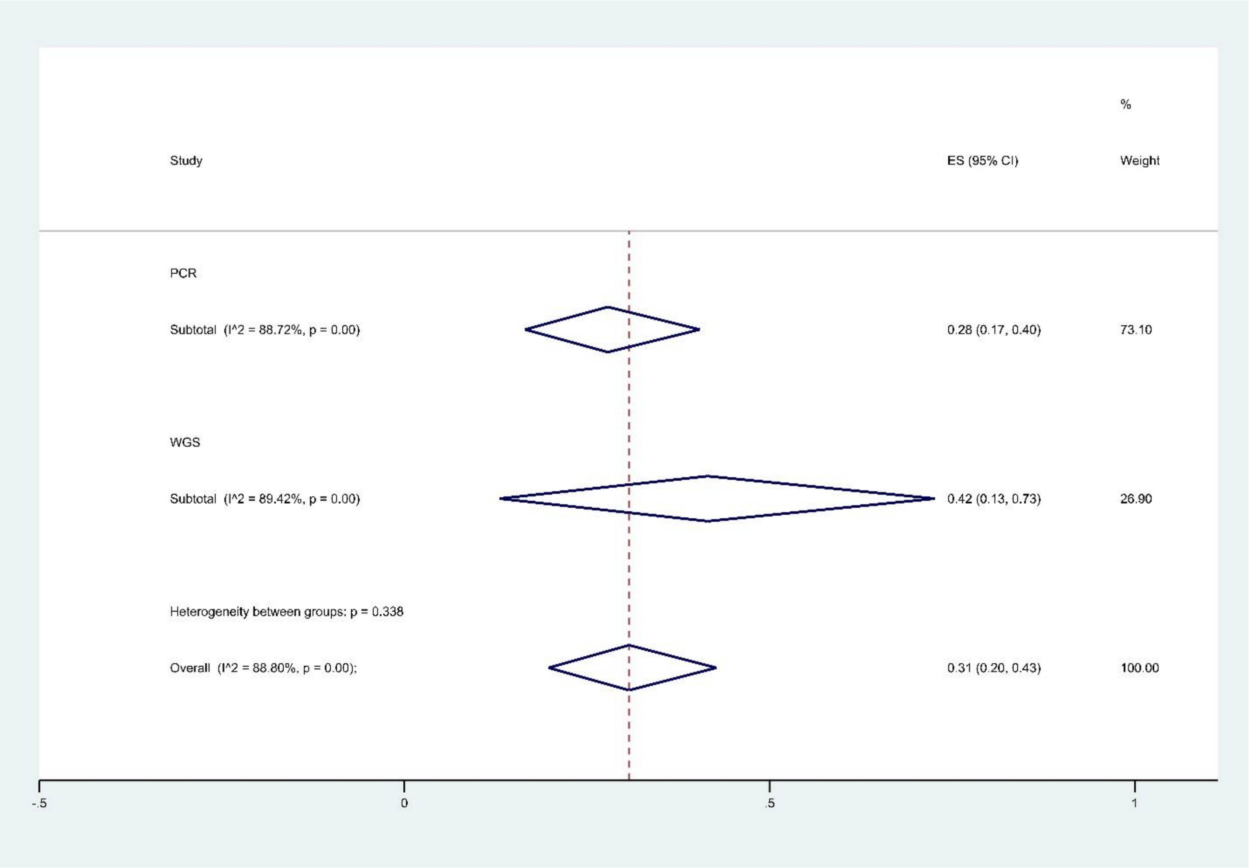

Figure 12: Subgroup meta-analysis for the method of nonsense mutation.

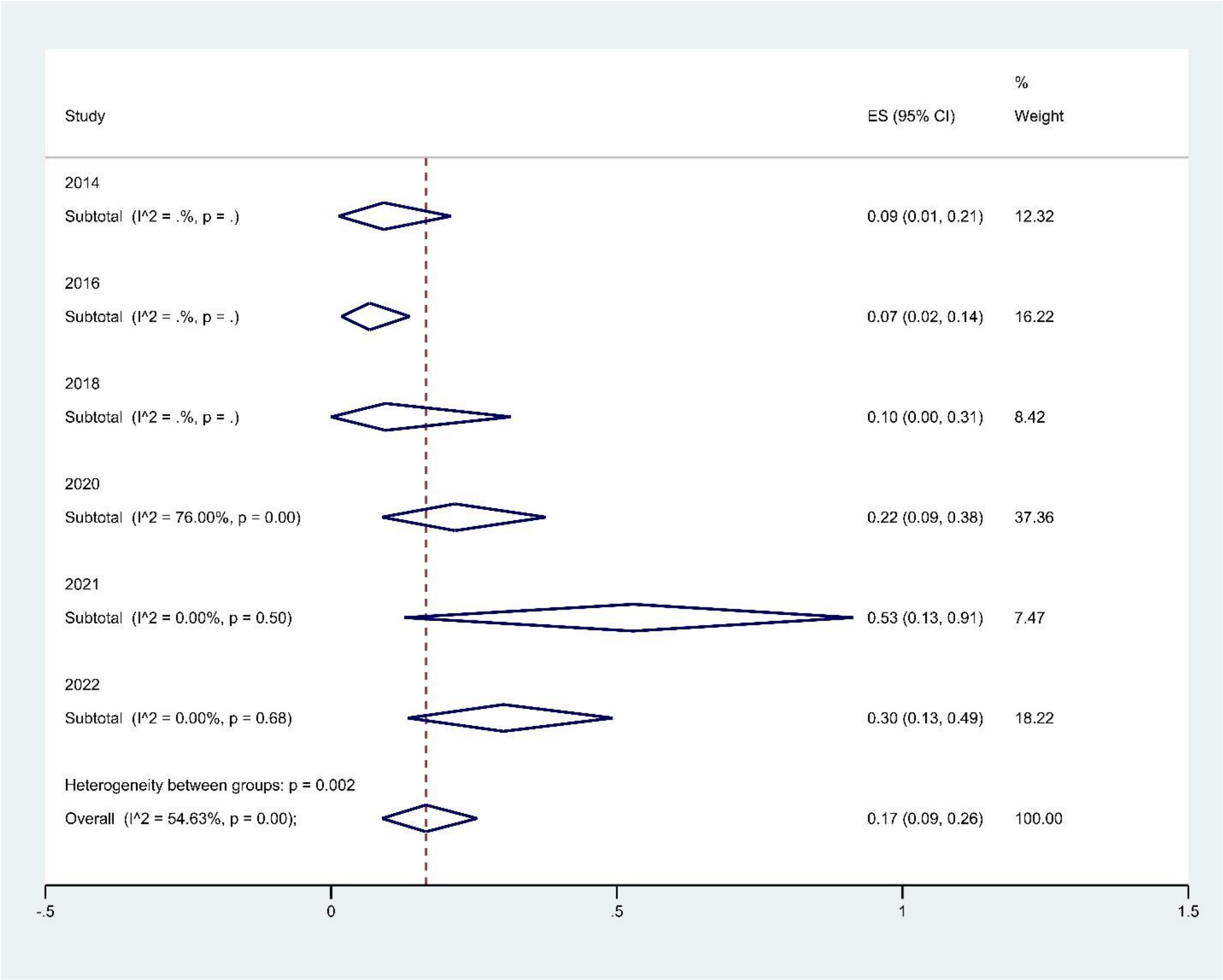

Figure 13: Subgroup meta-analysis for the year of complete deletion.

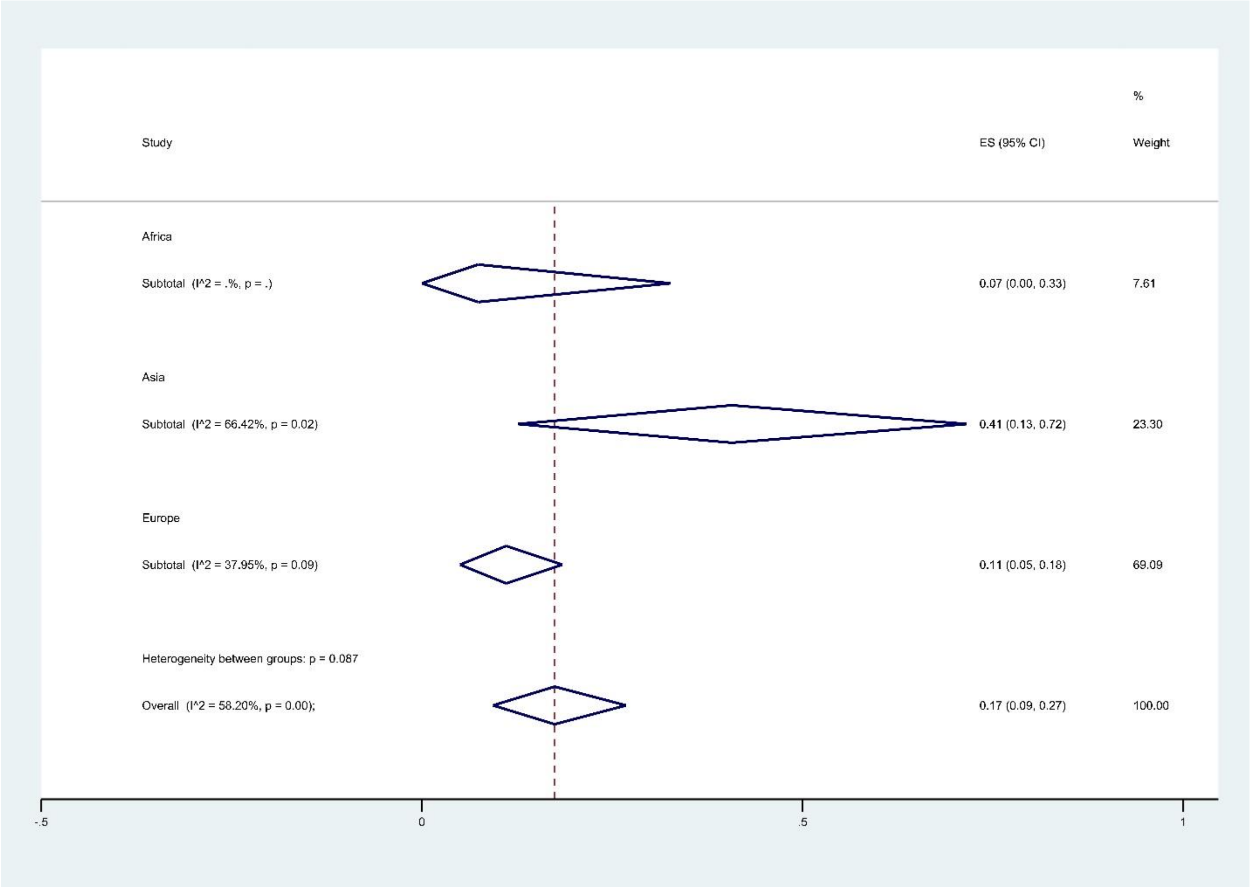

Figure 14: Subgroup meta-analysis for the continent of complete deletion.

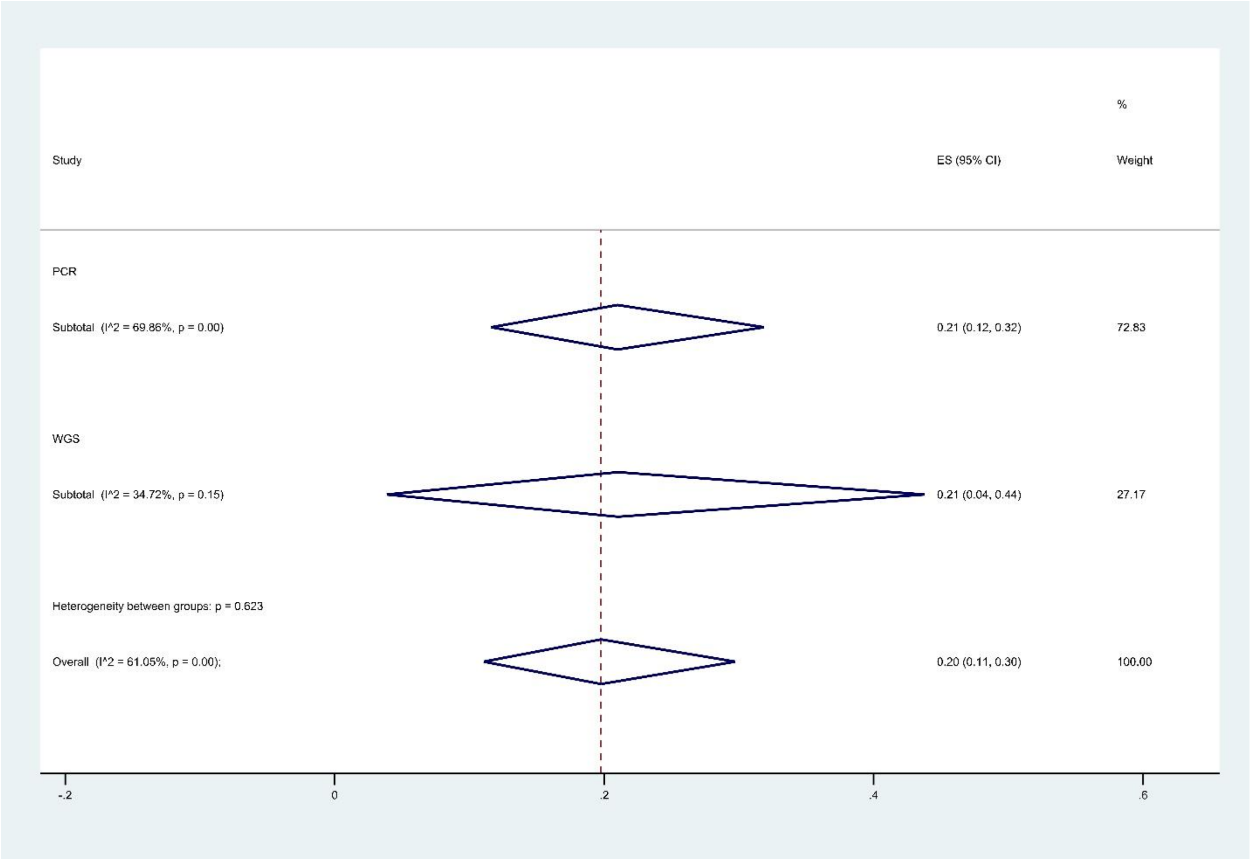

Figure 15: Subgroup meta-analysis for the method of complete deletion.

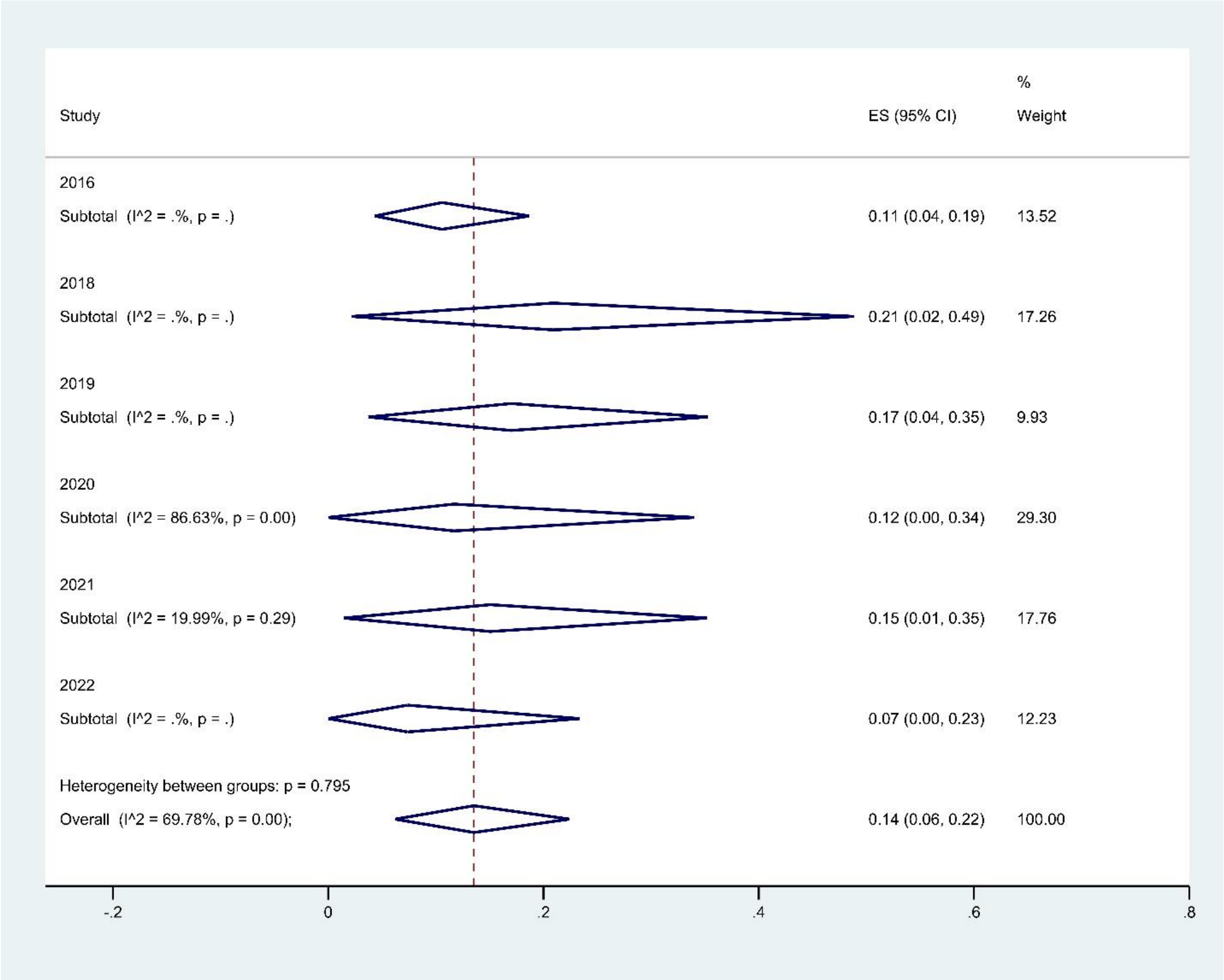

Figure 16: Subgroup meta-analysis for the year of partial deletion.

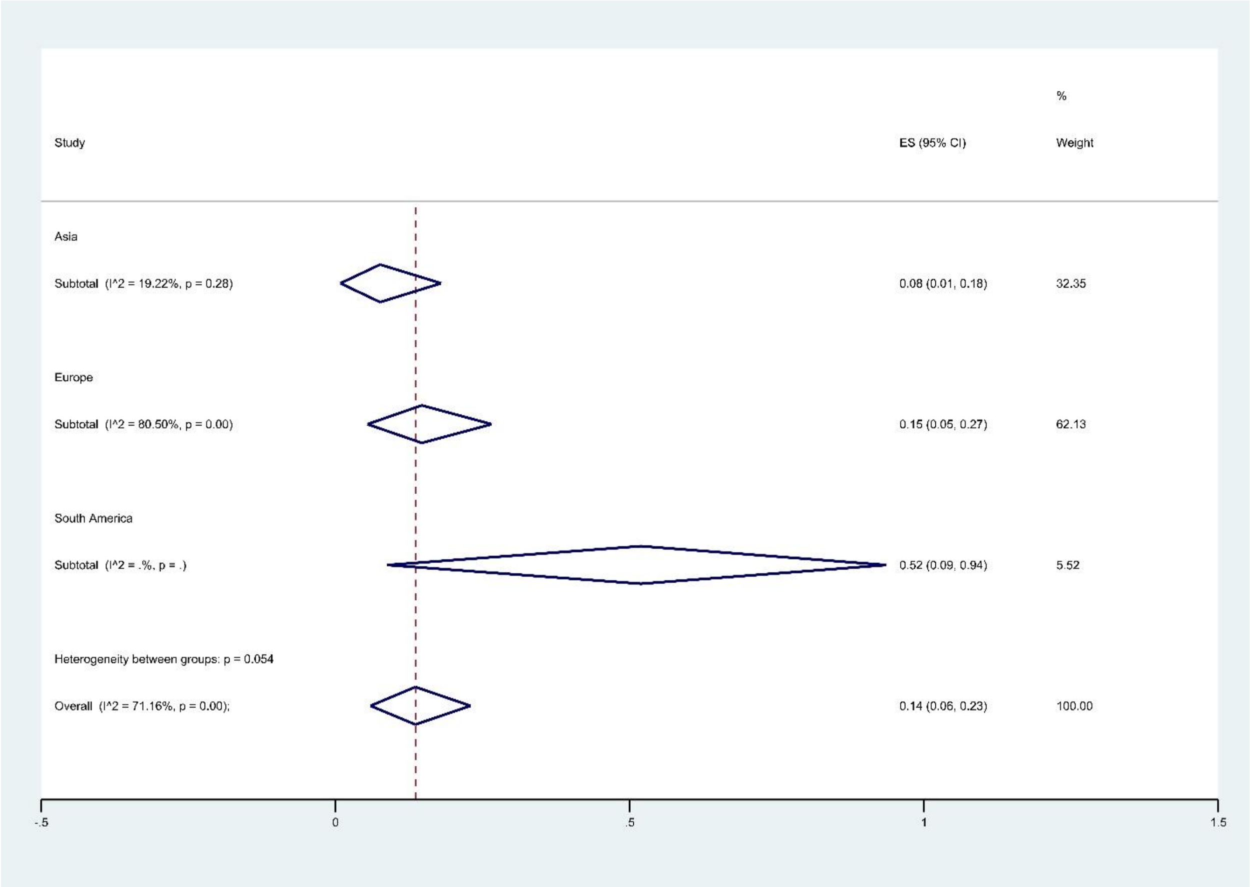

Figure 17: Subgroup meta-analysis for continent of partial deletion.

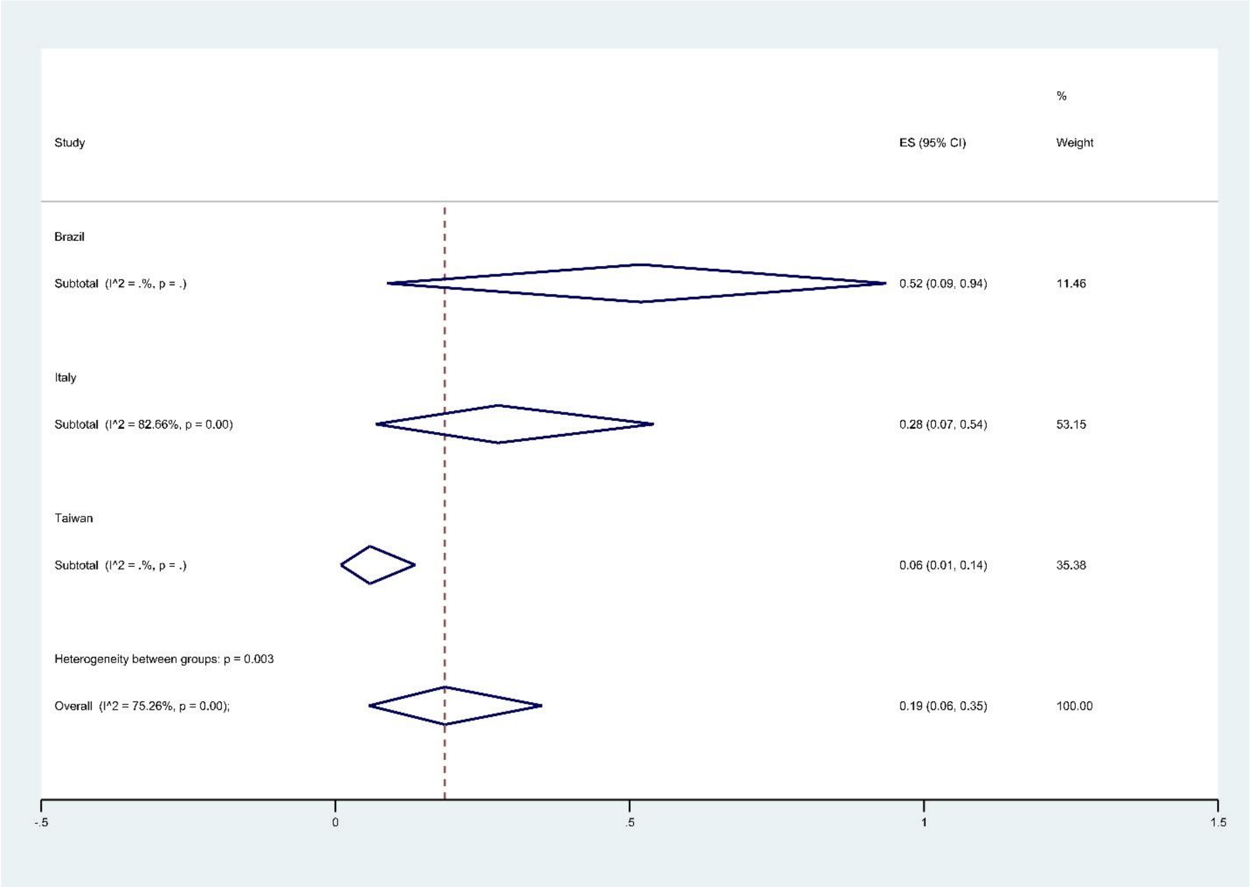

Figure 18: Subgroup meta-analysis for country of partial deletion.

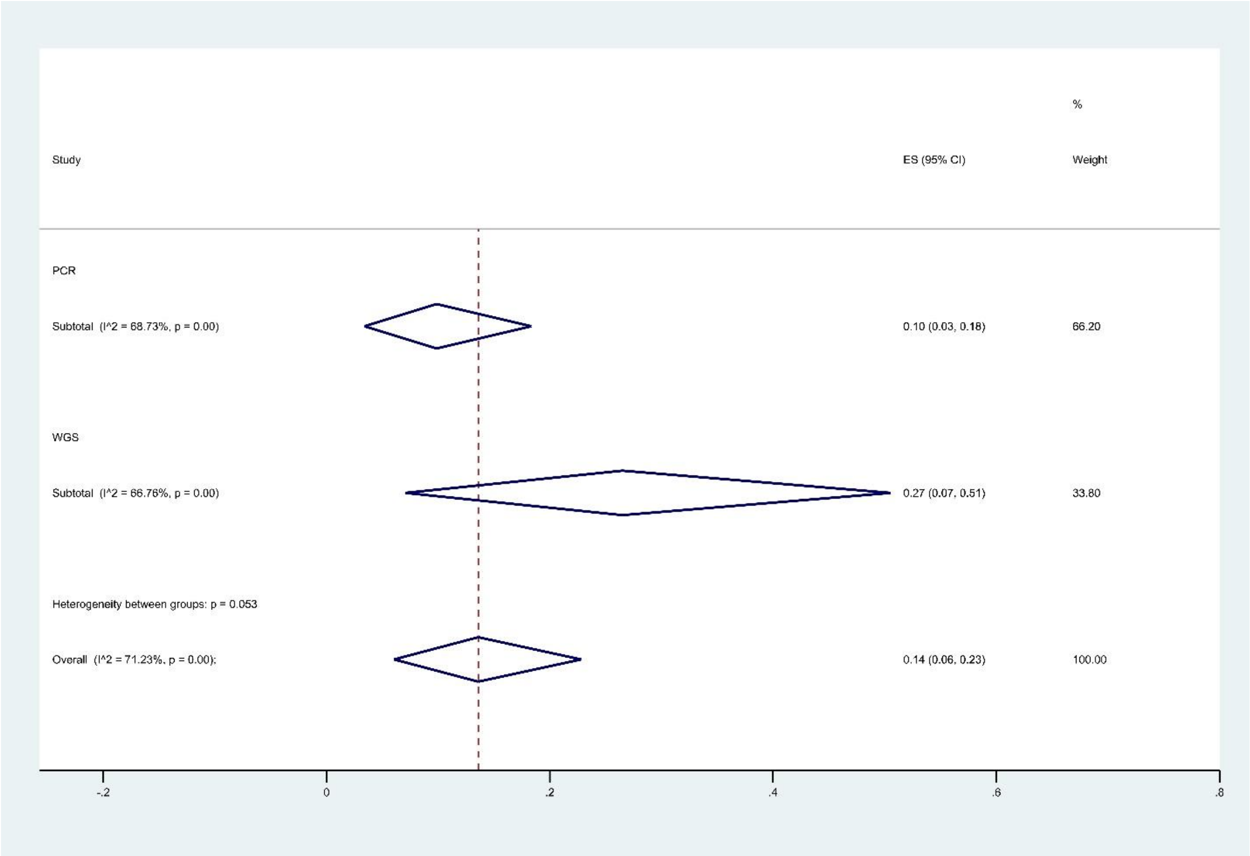

Figure 19: Subgroup meta-analysis for the method of partial deletion.
